# Single-cell microglial transcriptomics during demyelination defines a microglial state required for lytic carcass clearance

**DOI:** 10.1186/s13024-022-00584-2

**Published:** 2022-12-13

**Authors:** Sameera Zia, Brady P. Hammond, Martin Zirngibl, Anastasia Sizov, Charbel S. Baaklini, Sharmistha P. Panda, Madelene F. S. Ho, Kelly V. Lee, Apurba Mainali, Mena K. Burr, Sioned Williams, Andrew V. Caprariello, Christopher Power, Thomas Simmen, Bradley J. Kerr, Jason R. Plemel

**Affiliations:** 1 Neuroscience and Mental Health Institute, University of Alberta, Edmonton, Canada; 2 Department of Clinical Neurosciences, Hotchkiss Brain Institute, University of Calgary, Cumming School of Medicine, Calgary, Canada; 3 Department of Medicine, Division of Neurology, University of Alberta, Edmonton, Canada; 4 Department of Medical Microbiology and Immunology, University of Alberta, Edmonton, Canada; 5 Department of Cell Biology, University of Alberta, Edmonton, Canada; 6 Department of Anesthesiology & Pain Medicine, University of Alberta, Edmonton, Canada

**Keywords:** Microglia, Demyelination, Cuprizone, Phagocytosis, Single-cell RNA sequencing, Heterogeneity, Primary culture

## Abstract

**Background:**

Microglia regulate the response to injury and disease in the brain and spinal cord. In white matter diseases microglia may cause demyelination. However, how microglia respond and regulate demyelination is not fully understood.

**Methods:**

To understand how microglia respond during demyelination, we fed mice cuprizone—a potent demyelinating agent—and assessed the dynamics of genetically fate-mapped microglia. We then used single-cell RNA sequencing to identify and track the microglial subpopulations that arise during demyelination. To understand how microglia contribute to the clearance of dead oligodendrocytes, we ablated microglia starting at the peak of cuprizone-induced cell death and used the viability dye acridine orange to monitor apoptotic and lytic cell morphologies after microglial ablation. Lastly, we treated serum-free primary microglial cultures to model distinct aspects of cuprizone-induced demyelination and assessed the response.

**Results:**

The cuprizone diet generated a robust microglial response by week 4 of the diet. Single-cell RNA sequencing at this time point revealed the presence of several cuprizone-associated microglia (CAM) clusters. These clusters expressed a transcriptomic signature indicative of cytokine regulation and reactive oxygen species production with altered lysosomal and metabolic changes consistent with ongoing phagocytosis. Using acridine orange to monitor apoptotic and lytic cell death after microglial ablation, we found that microglia preferentially phagocytose lytic carcasses. In culture, microglia exposed to lytic carcasses partially recapitulated the CAM state, suggesting that phagocytosis contributes to this distinct microglial state during cuprizone demyelination.

**Conclusions:**

Microglia serve multiple roles during demyelination, yet their transcriptomic state resembles other neurodegenerative conditions. The phagocytosis of cellular debris is likely a universal cause for a common neurodegenerative microglial state.

**Supplementary Information:**

The online version contains supplementary material available at 10.1186/s13024-022-00584-2.

## Background

Multiple Sclerosis (MS) is a chronic inflammatory disease characterized by demyelinating lesions and ongoing neurodegeneration [1]. Lesions initially result from an infiltration of immune cells along with the expansion and activation of glial cells in the central nervous system (CNS) [2]. Within the lesion, immune cell dynamics are critical for tracking lesion evolution [3]. Of these, the innate immune cells known as microglia and macrophages predominate [4, 5]. Microglia are brain resident immune cells, while macrophages originate from monocytes or other brain resident macrophage populations such as meningeal macrophages [6, 7]. Microglia and macrophages are critical regulators of lesion formation and dynamics, and may potentially drive CNS demyelination [8]. Despite their importance, we do not yet know how these cells regulate ongoing demyelination.

Dietary consumption of cuprizone, a copper chelator, induces demyelination in the corpus callosum and cortical grey matter [9, 10]. Cuprizone-induced demyelination is not altered by the absence of lymphocytes and only involves the sparse infiltration of blood leukocytes [10, 11]. Intriguingly, the complete ablation of microglia prevents ultrastructural damage to myelin within the corpus callosum, suggesting that microglia are necessary for demyelination in this model [11]. Therefore, the cuprizone model is ideal for defining brain intrinsic mechanisms of demyelination. The grey matter demyelination, axonal degeneration, and microglial over-representation have led some to consider cuprizone toxicity as a model of the neurodegenerative aspects of progressive MS [12–14]. Understanding the mechanisms of how microglia participate in demyelination will be critical to finding strategies that limit ongoing neurodegeneration in MS.

Demyelination is linked to oligodendrocyte death; upon death, oligodendrocytes are subsequently cleared by phagocytic cells. Death of oligodendrocytes following the cuprizone diet is varied, with ongoing apoptosis [15, 16], necroptosis [17], ferroptosis [18], and, potentially, pyroptosis [19–21]. The causes of cell death after cuprizone consumption are likely diverse but may not involve the copper chelating properties of cuprizone [22]. It is unclear how the central nervous system’s phagocytic cells like microglia [23], oligodendrocyte progenitor cells [24, 25], and astrocytes [26–28] clear cellular carcasses resulting from these different forms of cell death. Here, we use microglial fate mapping, single-cell RNA sequencing, microglial ablation, and serum free microglial cultures to define the state and functions of microglia during demyelination. We identify a microglial state and find that it is essential for the clearance of dead cells, which, in turn, instructs this microglial state.

## Methods

### Animal information

We obtained CX3CR1^creER^ (JAX 021160), Ai9 (Rosa26^tdTom^; JAX 007905), Tmem119^creER^(JAX 031820), and Rosa26^iDTR^ (JAX 007900) mice from The Jackson Laboratory and bred locally. We used both male and female mice for these genetically modified mice lines. We obtained male C57/Bl6 mice (7-8 weeks) from Charles River. For genetic fate mapping, we used CX3CR1^creER^; Rosa26^tdTom^ (*n* = 23) and Tmem119^creER^;Rosa26^tdTom^ mice (*n* = 7), for single-cell experiments we pooled C57/Bl6 (10/group for cohort 1 and 15/group for cohort 2 for both naïve and 5 week cuprizone conditions, total *n* = 50), and for histological assessment of cuprizone timeline, we used C57/Bl6 (*n* = 32). For the microglia ablation experiment, CX3CR1^creER^; Rosa26^tdTom^; Rosa26^iDTR^ or littermate controls CX3CR1^creER^; Rosa26^tdTom^ mice were injected with saline or diphtheria toxin (*n* = 30). All groups, except CX3CR1^creER^;Rosa26^tdTom^, were fed a 0.25% cuprizone chow for 2 weeks prior to 10 days of ablation while keeping animals on the cuprizone chow. Fate mapping studies with CX3CR1^creER^;Rosa26^tdTom^ mice were conducted at the University of Calgary with 0.2% cuprizone and the remainder used 0.25% cuprizone and were conducted at the University of Alberta. For cell culture experiments, E13-E15 pregnant CD1 mice were ordered from Charles River. For animal experiments, all procedures were reviewed and approved by animal subcommittees at the University of Calgary and the University of Alberta.

### Animal treatments

#### Cuprizone

For studies at the University of Alberta, cuprizone was blended into standard mouse chow (PicoLab® Laboratory Rodent Diet 5L0D) at a concentration of 0.25% and administered to 6–8 week old mice based on pilot experiments where we determined the optimal dose to cause demyelination. For experiments with CX3CR1^creER^;Rosa26^tdTom^ mice, they were treated with 0.2% cuprizone, as previously described [29]. Animals were fed *ad labitum* until defined experimental endpoints.

#### Tamoxifen

CX3CR1^creER^;Rosa26^tdTom^, Tmem119^creER^;Rosa26^tdTom^, CX3CR1^creER^;Rosa26^tdTom^; Rosa26^iDTR^, or littermate control CX3CR1^creER^;Rosa26^tdTom^ were injected with 1 mg of tamoxifen (20 mg/ml; T5648, Sigma) dissolved in corn oil (C8267) daily for 3 days starting at postnatal day 12 or 13*.* Mice were placed on a cuprizone diet 4–6 weeks after tamoxifen injection.

#### Diphtheria toxin

Diphtheria Toxin (List Biological Labs, 150(LB)) was dissolved in 0.01 M Tris/0.001 M EDTA, pH = 7.5 to a concentration of 2 μg/ml and divided into 10 μl aliquots and stored at − 80 °C. For injections, mice were given 1 μg per day (10 μg/ml) for ten days. Saline control animals were injected with the same volume of 0.9% Sodium Chloride Solution (Hospira).

### Tissue processing

At the experimental endpoint, animals were euthanized with Euthanyl Reg (Pentobarbital Sodium, Vetoquinol, #127819) and transcardially perfused with PBS and 4% paraformaldehyde (PFA) in 0.1 M PB. Brains were removed, and post-fixed overnight with 4% PFA. Tissue was cryoprotected by placing it into 30% sucrose. Subsequently, brains were embedded in Tissue-Tek O.C.T. and snap frozen in liquid nitrogen.

### Immunohistochemistry

Brains were coronally sectioned into 20 μm sections with collection beginning at the most posterior aspect of the corpus callosum. Sections were collected onto Fisherbrand™ Superfrost™ Plus slides and stored at − 80 °C. Cryosectioned brain tissue was thawed, air dried for 30 minutes and placed into PBS for 10 minutes before being incubated for 1 hour with an antibody blocking solution of 10% Normal Donkey Serum (Sigma, 566,460), 0.1% Gelatin from cold water fish skin, 0.1% Triton X-100, 0.05% Tween-20 in 0.01 M PBS. Primary antibodies were diluted in Antibody dilution buffer containing 0.1% Gelatin from cold water fish skin and 0.1% Triton X-100 in 0.01 M PBS before incubating overnight at 4°. We used the following primary antibodies: rabbit anti-Olig2 (1:200, Millipore, AB9610(CH)), rat anti-Ki67 (1:200, Invitrogen, 14–5698-82), goat anti-IBA1 (1:500, Novus Biologicals, #NB100–1028), rabbit anti-IBA1 (1:1000, Wako, 019–19,741), rabbit anti-CD45 (1:150, Abcam, #ab10558), rat anti-CD45 (1:100 BD Pharmingen, #550539), rabbit anti-CD4 (1:200, Abcam, #ab183685), chicken anti-MBP (1:200, Aves lab, MBP (AVL)), rabbit anti-NINJ1 (1:200; ThermoFisher Scientific PA5–72821), rabbit anti-cleaved caspase-3 (1:100; Cell Signaling Technology, no.9661S), rat anti-Ly6G (1:200, Biolegend, #127601), rat anti-Dectin1 (1:50, Invivogen, #madg-mdect). Slides were washed 3–5 times with PBS containing 0.5% Tween 20, before incubating for 2 hours at room temperature with secondary antibodies diluted in the antibody dilution buffer. We used anti-rat, −mouse, −rabbit, −goat Alexa Fluor 488, 594, or 647 conjugated F(ab)_2_ fragment secondary antibodies (1:400, Jackson ImmunoResearch). Slides were also stained with DAPI. Slides were washed 3–5 times with PBS + 0.5% Tween 20 and mounted with Fluoromount G. Images were collected with either a Leica SP5 or a Leica SP8 confocal microscope. We verified that bleed-through was negligible.

### Tissue staining

For Eriochrome Cyanine (EC staining), tissue sections were thawed and treated with a sequence of 2 min in CitriSolv I (Fisher Scientific, 04–355-121), 1 minute in isopropanol, decreasing concentrations of ethanol (100, 95, 85, 70, 50%; all 1 minute), and 1 minute dH_2_O. Slides were incubated for 15 minutes in EC (0.16%, Millipore-Sigma, #1.03164) and then differentiated for ~ 10 seconds with 0.5% aqueous NH_4_OH, and washed in dH_2_O. Slides were stained additionally with 1% neutral red solution (1%, Fisher Scientific, 553–24-2) for 2 minutes. Slides were then washed for 1 minutes in dH_2_O, dehydrated with ascending concentrations of ethanol (50, 70, 85, 95, 100%; all 1 minute), before incubating them in isopropanol for 2 minutes and CitriSolv I twice for 2 minutes each. Slides were then mounted with Acrytol. For acridine orange (AO) staining, frozen tissue was air dried for 30 minutes and washed with 1x RNase free PBS for 10 minutes. Subsequently, sections were stained with AO (50 μM, Invitrogen, A3586) and DAPI (5 μg/ml, Invitrogen, #1306) in RNase free PBS for 20 minutes. The AO solution was removed and slides were mounted with Fluoromount G. For Immunohistochemistry following AO stain, slides were incubated in 1x PBS for 1h until mounted coverslip came off easily. The tissue sections were subsequently washed 2 times 5 minutes with PBS to remove Fluoromount G, and 3 times 5 minutes with PBS + 0.05% Tween 20 to removed RNA bound AO. Thereafter, the immunohistochemistry outlined above was followed starting from the antibody blocking step. Images were acquired at a Leica SP8 or a Leica SP5 Confocal with a single excitation at 488 nm and emission collection at 490–550 nm (green) and 580–750 nm (red).

### Single cell RNA sequencing

#### Fluorescence activated cell sorting

Mice were euthanized with Euthanyl Reg and transcardially perfused with ice-cold HBSS (Invitrogen 14175103). Brains were dissected, processed, and diced in ice-cold HBSS. Collected tissue was then dounced 5 times with a loose fitted dounce and subsequently filtered through 70 and 20 μm cell strainers. Cells were pelleted and myelin debris was removed with Debris Removal Solution (Miltenyi Biotech 130–109-398). Cells were then blocked for 5 minutes with Anti-Mo CD16/CD32 (Invitrogen 14–0161-85) and stained using Zombie Aqua Fixable Viability Kit (Biolegend 423102), DRAQ5 Fluorescent Probe Solution (Thermofisher 62254) and FITC Mouse anti Rat CD11b (BD Biosciences 561684) for 20 min. After cell staining, cells were sorted on a Sony MA900. Live nucleated single cells that were CD11b positive were gated and collected. Final collected cell counts were determined using a hemocytometer.

#### Sequencing preparation

The FACS-isolated cells were processed for single cell sequencing on the 10x Genomics Chromium Controller (PN-1000121) using the 10x Genomics Chromium Next GEM Single Cell 3′ GEM, Library and Gel Bead Kit v3.1 (PN-1000121), following the manufacturer’s instructions and sequenced on the Illumina HiSeq P150 sequencer (Novogene). We chose the paired end, single indexing form of sequencing with an average read depth of 50,000 reads per cell. The BCL files generated from the sequencer were demultiplexed into FASTQ files and aligned to a custom *Mus musculus 10* (mm10) reference genome specifically designed to include the polymorphic psuedogene, *Clec7a*. This was done by adding additional annotations of *Clec7a* from the 10x parent mm10 reference GTF file to the 10x-provided mm10 GTF reference genome file. The final genome was created by combining the mm10 reference FASTA file with the custom GTF file using the 10x cell ranger (v3.0.0) pipeline, specifically the mkref function. The samples were aligned to the custom genome using the cell ranger count function thereby generating barcoded and sparse matrices.

#### Quality control and clustering

All the quality control was performed using Seurat v4.0 [30, 31] (https://github.com/satijalab/seurat) in the R statistical environment (v4.1.2). A Seurat object was created using the CreateSeuratObject() function to include genes expressed in a minimum of 3 cells and cells expressing a minimum of 200 genes. The object was further subsetted using the subset() function to remove doublets or multiplets—cells with high gene counts (> 3000) and dead cells—and cells with high percentages of mitochondrial genes (> 10%). All datasets were down-sampled to 1000 cells, using the subset() function and merged with the merge() function. The SCTransform() function (Highly variable features = 3000), was used to normalize the merged data according to the binomial regression model [32].

Dimensionality reduction was performed using RunPCA(), FindNeighbours() and FindClusters() functions successively while retaining 25 PCs, as determined by a PCA elbow plot. The dataset was clustered at successive resolutions separated by 0.1 by varying the resolution parameter (0–1) in the FindClusters() function. The final resolution of 0.5 was selected based on the clustering tree generated from the Clustree package [33]. The clustree() function was used to create a tree containing clusters present at all resolutions (0–1, as determined by FindClusters() function), and the resolution of 0.5 was chosen based on the most stable level as visualized by the tree.

The dataset was converted to an h5ad file using SeuratDisk and loaded into a python environment (Python 3.8.3) of a Jupyter notebook (v6.0.3) using Scanpy (v1.6.0) [34]. The cells were further refined using the Single Cell Clustering Assessment Framework (SCCAF) package (v0.0.10, https://github.com/SCCAF/sccaf) [35] where a machine learning algorithm iteratively generated clusters (150 iterations) until a 95% self-projection accuracy was reached. The final clustering iteration was projected onto a UMAP.

#### Gene set comparisons

The gene sets were calculated by comparing the control and cuprizone libraries. The up- and down- regulated differentially expressed genes (DEGs) of each library were calculated using the FindAllMarkers function in Seurat (min.pct = 0.3, logFCthreshold = 0.3), retaining 227 genes (*p* < 0.05). The Euclidean distance between the DEGs was calculated using the dist() function, and the DEGs were further clustered using Ward’s methods (ward. D2) in the hclust() function of the stats R package (v4.1.2). The branches were divided into 4 gene sets using the cutree() function and the average gene expression values were plotted in Seurat using the DoHeatmap() function.

#### Gene regulatory network analysis

We assessed gene regulatory networks using the python-based Single-Cell Regulatory Network Inference and Clustering pipeline (pySCENIC, v0.10.0, https://github.com/aertslab/pySCENIC). The genome ranking databases and transcription factor annotations were downloaded from cisTargetDBs (https://resources.aertslab.org/cistarget/), specifically the *Mus Musculus* 9 (500 base pairs upstream of the transcription start site; Motif collection V9; 7 orthologous species) and *Mus Musculus* 9 (10k bases around the transcription start site for a total of 20k bases; Motif collection V9; 7 orthologous species) databases and Mouse TFs (Motif collection V9). The co-expression modules were determined using the pyscenic grn function (seed = 42). The co-expression modules were pruned using the CisTarget databases specified above in the pyscenic ctx function (num_workers = 20). The regulon—network of transcription factors, regulators, and targets—activity was scored using the pyscenic aucell function (num_workers = 20). The regulon activity was integrated with the overall sequencing data using the add_scenic_metadata() function and the regulon specificity score for each cluster was calculated using the regulon_specificity_scores() function. The top 2 regulons per cluster were plotted using the sns.heatmap() function. Finally, the individual regulons were explored in Cytoscape using the iRegulon plugin.

#### Receptor-ligand interactions

We assessed receptor-ligand interactions using the R-based intercellular communication tool, Nichenet [36] (v1.0.0, https://github.com/saeyslab/nichenetr). Specifically, we compared the ligands released from both saline-treated (GSM4475128) and LPS-treated (GSM4475134) astrocytes from a publicly available dataset (GSE148612) [37] with respect to the receptors present on CAMs1–3. The ligand-target matrix (ligand_target_matrix.rds), ligand-receptor network (lr_network.rds) and weighted networks (weighted_networks.rds) were downloaded from Zenodo (https://zenodo.org/record/3260758#.Y2U9wezMKw4). The genes expressed by astrocytes and microglia were extracted using the get_expressed_genes() function (min.pct = 0.10) and filtered to keep only those present in the ligand-receptor network (ligands-astrocytes, receptors-microglia). The markers expressed by the CAMs relative to the astrocytes were isolated using the FindMarkers() function (ident.1 = CAMs, ident.2 = astrocytes, min.pct = 0.10) and filtered to include genes with adjusted *p*-value <=0.05 and absolute value of log threshold > 0.25. The genes present in the ligand-target matrix were retained. The predicted ligands activity was calculated using the predict_ligand_activities() function and was sorted in descending order of Pearson correlation. The top-ranked genes were isolated from the weighted networks and plotted on a heatmap.

#### RNA velocity

RNA velocity was calculated using the ScVelo (v0.2.2, https://github.com/theislab/scvelo) package [38]. The spliced and unspliced gene abundancies were recovered using the tl.recover_dynamics() and tl.velocity() functions (mode = dynamical). The velocity for cells was plotted in stream lines using the pl.velocity_embedding_stream() function. The genes with high velocity differentials were calculated using the tl.rank_velocity_genes() function (Min_corr = 0.3) and the genes were plotted using the pl.heatmap() function. Further the initial and terminal states were defined using CellRank (v1.0.0, https://github.com/theislab/cellrank) [39] using the tl.initial_states() and tl.terminal_states() (weight_connectivities = 0.2) functions respectively.

#### Functional gene ontology terms

The DEGs per cluster were calculated using the FindAllMarkers() function (min.pct = 0.3, logFCthreshold = 0.3). The top 50 DEGs (*p* < 0.05) were inputted into the gProfiler web server (https://biit.cs.ut.ee/gprofiler/gost) to generate functional Gene Ontology terms, specifically from the gene ontology (molecular function, cellular component, and biological processes), KEGG and REACTOME databases. Terms with a *p* value < 0.05 were retained and plotted on an alluvial plot using the ggalluvial package (v0.12.3) where the thickness of the bands corresponds to the genes at the intersection of the terms and clusters. The genes associated with each term were averaged and visualized through density plots using the plot_density function of the Nebulosa package (v1.4.0) [40].

### Cell culture

#### Oligodendrocyte culture and lysis 

OPCs were isolated via immunopanning as previously described [41]. Briefly, we dissected cortices from postnatal day 5–7 CD1 mice and dissociated with the gentleMACs Neural Tissue Dissociation Kit P (Miltenyi Biotec 130–092-628). Dissociated tissue was passed over two *Griffonia (Bandeiraea) simplicifolia* lectin-1 (BSL-1) (Vector Laboratories VECT1100) coated dishes for 15 minutes each to remove microglia, macrophages, and endothelial cells, followed by placing the resultant cell suspension in an anti-GalC coated dish [42] for 45 minutes to remove post-mitotic oligodendrocytes. The cell suspension was lastly placed into an anti-O4 coated dish [43, 44] for 45 minutes to positively select for OPCs. OPCs were removed from the plate by exposing cells to a trypsin solution (50,000 units/ml; Sigma-Aldrich T9935) for seven minutes followed by repeated pipetting to dislodge cells. Viable cells were counted and plated between 200,000–500,000 cells/plate on poly-D-lysine (10 mg/ml; Sigma-Aldrich P6407) coated tissue culture plates in OPC proliferation media. OPC proliferation media and differentiation media contained the same base of DMEM (Gibco 11960069), apo-transferrin (100 μg/ml; Sigma-Aldrich T1147), bovine serum albumin (100 μg/ml; Sigma-Aldrich A4161), putrescine dihydrochloride (16 μg/ml; Sigma-Aldrich P5780), progesterone (60 ng/ml; Sigma-Aldrich P8783), sodium selenite (40 ng/ml; Sigma-Aldrich S5261), insulin (5 μg/ml; Sigma-Aldrich I6634), GlutaMax (2 mM; Gibco 35050–061), PenStrep (1000 units/ml; Gibco 15140–122), sodium pyruvate (1 mM; Gibco 11360–070), n-acetyl cysteine (5 μg/ml; Sigma-Aldrich A8199), trace elements B (1x; Corning MT99175CI), B27+ supplement (2x; Gibco A3582801), and d-biotin (10 ng/ml; Sigma-Aldrich B4639). For OPC proliferation, PDGF-AA (10 ng/ml; Peprotech 100-13A), NT3 (10 ng/ml; Peprotech 450–03), CNTF (10 ng/ml; Peprotech 450–13) and forskolin (4.2 μg/ml; Sigma-Aldrich F6886) were added to base media. OPCs were expanded to ~ 80% confluency and differentiated with OPC differentiation media where PDGF-AA was removed and T3 (40ng/ml; Sigma-Aldrich T6397) was added.

OPCs were differentiated into oligodendrocytes by removing mitogens for 48 hours. Oligodendrocytes were placed into base media for one hour and then incubated with lysophosphatidylcholine (100 μM; Sigma-Aldrich L1381) for one hour at 37 °C. We collected both conditioned media and scraped the dish to obtain oligodendrocyte lytic cell carcasses. All media conditions were spiked with bovine serum albumin (2%; ThermoFisher 11,020,021) and stored at 4 °C until use.

#### Microglia culture, live imaging and immunocytochemistry

Microglia were isolated via immunopanning and cultured in serum-free conditions as previously described [45]. Briefly, we dissected cortices from postnatal day 5–7 CD1 mice (Charles River) and dissociated cortical tissue with the gentleMACs Neural Tissue Dissociation Kit P (Miletnyi Biotec 130–092-628). Dissociated tissue was incubated in an anti-CD11b (clone M1/70, Invitrogen 14–0112-85) coated dish to positively select microglia. Microglia were trypsinized (30,000 units/ml; Sigma-Aldrich T9935) for 10 minutes, dislodged via repeated pipetting, counted, and plated in serum-free growth media on poly-d-lysine coated (10 mg/ml; Sigma-Aldrich P6407) 96-well culture plates at a density of 10,000 microglia/well. Microglia growth media was prepared with a Neurobasal Media base (Gibco 21103049), Gem21 supplement (1x; GeminiBio 400–160-010), N2 supplement (1x; ThermoFisher 17502–048), lipated BSA (1 mg/ml; ThermoFisher 11,020,021), GlutaMax (2 mM; Gibco 35050–061), sodium pyruvate (1 mM; Gibco 11360–070), sodium chloride (50 mM; Sigma-Aldrich S5150), d-biotin (10 ng/ml; Sigma-Aldrich B4639), and lactic acid (0.2%; Sigma-Aldrich L1250). Microglia were supplemented with colony-stimulating factor 1 (10 ng/ml; Peprotech 315–02), transforming growth factor β 2 (2 ng/ml; Peprotech 100-35B), cholesterol (1.5 μg/ml; Avanti Polar Lipids 700000P), heparan sulfate (1µg/ml; Galen Laboratory Supplies GAG-HS01), oleic acid (100ng/ml; Cayman Chemicals 90260), and gondoic acid (1ng/ml; Cayman Chemicals 20606) to support survival in serum-free conditions. 

For assessments of in vitro microglial phagocytosis, microglia were cultured for five days before treatments. For live-imaging experiments, CellTracker Green (10 μM, ThermoFisher C7025) and NucBlue™ (4 drops/ml, ThermoFisher R37605) were diluted in microglia media and incubated for 45 minutes at 37 °C. To track phagocytosis, CellTracker/NucBlue media was removed and microglia were exposed to myelin (5 μg myelin/well) and nuclei (10,000 nuclei/well) labeled with pHrodo™ Red (Invitrogen P36600), according to the product protocol. For certain phagocytosis studies, microglia were also exposed to 10% FBS media, cytochalasin D (10 μg/ml) dissolved in DMSO (Millipore-Sigma C2618), or equivalent volume DMSO. Upon treatment, microglia were immediately transferred to an ImageXpress Pico (MolecularDevices) for live imaging.

To study Dectin1 expression, microglia were cultured for five days before being treated with nuclei (10,000 nuclei/well), myelin (5µg myelin/well), oligodendrocyte-conditioned media diluted 1:1 in microglia media, lipopolysaccharide (LPS) (1 μg/ml), phosphatidylserine (1 μg/ml; Avanti Polar Lipids 840032P), IL-1β (10 ng/ml; R&D Systems 401-ML-005), TNF-α (10 ng/ml; R&D Systems 410-MT). After 72 hours, microglia were fixed with 4% paraformaldehyde, blocked with 10% normal donkey serum (Sigma-Aldrich D9663) diluted in 0.1% Triton-X100 and Dulbecco's phosphate buffered saline (DPBS) with calcium and magnesium (Sigma-Aldrich D8662) for 45 minutes at room temperature. Then cells were incubated with primary antibody solution containing rat anti-mouse Dectin1 (2 μg/ml; InvivoGen mabg-mdect) and rabbit anti-mouse CD11b (5 μg/ml; Novus Biologicals NB110–89474) overnight at 4 °C. Cells were washed with DPBS and incubated with secondary antibody solution containing anti-rat 488 (3.75 μg/ml; Jackson ImmunoResearch 712–546-153), anti-rabbit Cy3 (3.75 μg/ml; Jackson ImmunoResearch 711–166-152), and DAPI (5 μg/ml; Invitrogen D1306) for two hours at room temperature. Microglia were washed with DPBS and imaged using the ImageXpress Pico (Molecular Devices). All analyses were conducted in CellReporterXpress using the multiwavelength cell scoring analysis tool.

### Statistics

To determine statistical significance (*P < 0.05) we used either a one or two-way analysis of variance (ANOVA) with post hoc testing (details in figure legends) with Prism Software (GraphPad by Dotmatrics). All data were presented as means ± SEM.

## Results

### Microglia predominate within the corpus callosum during cuprizone demyelination

Dietary cuprizone induces demyelination three to four weeks after mice begin a cuprizone diet [22]. Similarly, the population of microglia and monocyte-derived macrophages begin to expand after two weeks and peak at roughly six weeks based on the expression of non-specific microglia and macrophage markers [22, 46]. We first wanted to understand what myeloid cells respond to cuprizone demyelination. To define the microglial response, we used a tamoxifen-inducible cre-recombinase dependent reporter system under the CX3CR1 promoter [47, 48]. With these mice, tamoxifen induces tdTomato expression in myeloid cells, including microglia, peripheral monocytes, and border-associated macrophages (BAMs), which include meningeal, perivascular and choroid plexus populations [49].

**Fig. 1:**
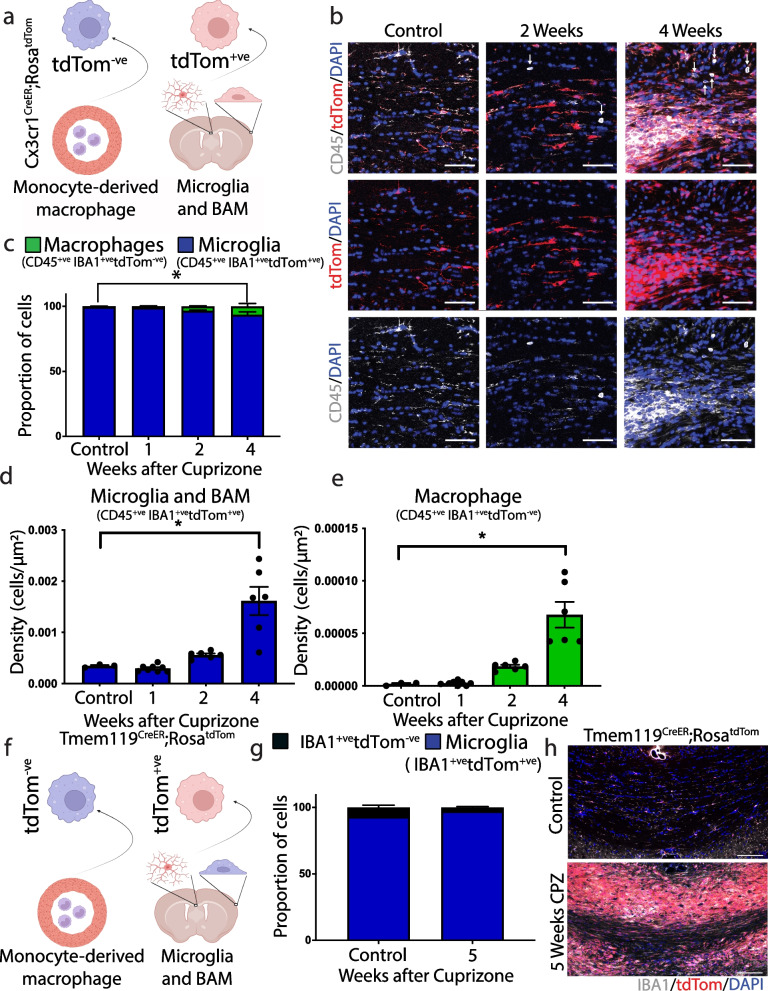
Microglia predominate within the corpus callosum following cuprizone induced demyelination. **a** Schematic of myeloid cells in CX3CR1^creER^;Rosa26^tdTom^ mice 4 weeks post-tamoxifen. The tdTomato (tdTom) is restricted to microglia and border associated macrophages (BAM). **b** Representative immunohistographs of CX3CR1^creER^;Rosa26^tdTom^ mice on a control, two, or four week, cuprizone diet demonstrating preferential microglia expansion (tdTom reporter, red; CD45, white). Quantification of the proportion (**c**) and density (**d**) of microglia/BAM (IBA1^+ve^/CD45^+ve^/tdTom^+ve^) and (**e**) monocyte-derived macrophages (IBA1 + ve/CD45^+ve^/tdTom^-ve^) show preferential expansion of microglia/BAM. **f** Schematic of myeloid cells in Tmem119^creER^;Rosa26^tdTom^ mice. Quantification of the proportion (**g**) of microglia (IBA1^+ve^/tdTom^+ve^) and (**h**) representative images confirm preferential expansion of microglia after cuprizone. Arrows indicate tdTom^-ve^/CD45^+ve^ cells. (**c-e**) *n* = 3 (control) or *n* = 6–8 (1,2,4 week after cuprizone); Ordinary one-way ANOVA with Tukey’s multiple comparisons test (*p* < 0.05). Error bar ± SEM. (**b**) Scale bar, 50 μm

We gave mice tamoxifen starting at postnatal days 12–13, which labels virtually all microglia and macrophages and waited 4–6 weeks. At this point, only tissue-resident macrophages such as microglia and BAMs retain the expression of tdTomato [49, 50]. We then placed mice on a cuprizone diet and counted tdTomato expressing microglia and BAMs before (1 and 2 weeks) and after (4 weeks) demyelination (Fig. 1a, b). Overall, we found that the vast majority of IBA1 (myeloid marker) and CD45 (pan-leukocyte marker) labelled cells expressed tdTomato, indicating that they were microglia or BAMs (Fig. 1b, c). We examined the BAM marker Lyve1 [51] to determine if BAMs contribute to cuprizone-induced demyelination and found Lyve1 labelled cells did not migrate into the corpus callosum during demyelination (Supp Fig. 1a). Microglial numbers roughly doubled between 2 and 4 weeks after cuprizone (Fig. 1d), concordant with ongoing demyelination. Microglia expressed the proliferative marker (Ki67) suggesting microglia proliferate between two and  four weeks after cuprizone (Supp Fig. 2a), consistent with the findings of others [52]. At the same timepoints, we found microglia contain myelin basic protein (MBP), indicating that microglia phagocytose myelin debris (Supp Fig. 2b).

We found a smaller population of IBA1 and CD45 labelled cells that did not express tdTomato, 3.3% at two weeks and 5.2% at four weeks on the cuprizone diet (Fig. 1c, e). Given that IBA1 can be found on T-cells and monocytes, we examined whether tdTomato-negative cells were T-cells using a T-cell specific marker, CD4. We found that a small population of these non-tdTomato CD45 labelled leukocytes express CD4, suggesting a portion of them were T-cells and the remainder were likely monocyte-derived macrophages that infiltrate the brain parenchyma (Supp Fig. 1b-e). Similarly, we found no neutrophils within the corpus callosum parenchyma following dietary cuprizone (Supp Fig. 1f, g). To examine the microglial origin of IBA1-positive immune cells in the corpus callosum using a different Cre-line, we injected Tmem119^CreER^;Rosa26^TdTom^ mice with tamoxifen, which induces tdTomato expression in microglia, but not monocytes or BAMs [53] (Fig. 1f). For mice on a control diet ~ 95% of microglia expressed tdTomato, suggesting a minor portion of microglia did not undergo Cre-mediated recombination (Fig. 1g, h). Using Tmem119^CreER^;Rosa26^TdTom^ mice we confirmed that the vast majority of IBA-expressing cells in the corpus callosum during demyelination were microglia. Taken together, dietary cuprizone promotes microglial expansion and recruits a small population of T cells and monocyte-derived macrophages into the corpus callosum.

### Distinct microglial state present during cuprizone-induced demyelination

**Fig. 2:**
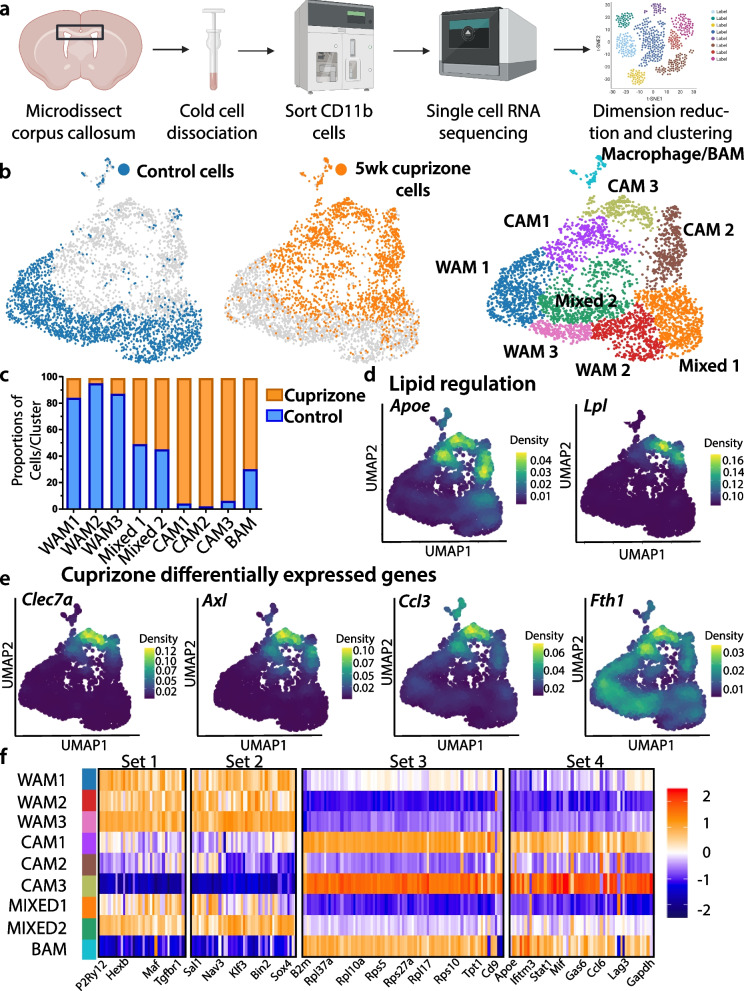
Single-cell RNA sequencing of corpus callosum myeloid cells from naïve mice and mice on a five week cuprizone diet. **a** Schematic showing microdissections, dissociation, fluorescence activated cell sorting (FACS), single cell RNA sequencing, and bioinformatic workflow. **b** Unsupervised graph-based clustering and machine learning-based reclustering of dataset projected onto a Uniform Manifold Approximation and Projection (UMAP). Most microglia isolated from naïve mice (control cells, blue) segregate from microglia isolated from cuprizone fed mice (orange) demonstrating distinct microglial states. **b, c** Microglia segregated into three cuprizone associated microglia (CAM) clusters, three white matter associated microglia (WAM) clusters enriched with homeostatic cells, and two clusters with a mixture of cuprizone and control cells. **d, e** Kernel density estimates of genes related to lipid regulation (*Apoe* and *Lpl*) and inflammation (*Clec7a*, *Axl*, *Ccl3*, and *Fth1*) were projected onto UMAPs and were found to be enriched within CAM. **f** Hierarchical clustering of differentially expressed genes with Wards method produced four gene sets including 227 genes that could differentiate clusters. Legend represents average gene expression values with red and blue indicating higher and lower expression, respectively

During pathological states, microglia respond in complex and incompletely understood ways. Microglia may tailor their response to the unique features of different pathologies. Indeed, the microglial state in the aged white matter differs from the microglial state during amyloid-associated neurodegeneration [54–56]. Currently, the properties of microglia during cuprizone-induced demyelination are not well characterized. To define the microglial response to cuprizone-induced demyelination, we conducted single-cell RNA sequencing (scRNAseq) as this is a powerful approach to understand disease-associated transcriptional changes within individual cells [54, 57–60]. We used fluorescence-activated cell sorting (FACS) to isolate CD11b-expressing cells from the corpus callosum of control mice or mice fed a cuprizone diet for five weeks (Fig. 2a). We conducted droplet-based scRNAseq, discarded low-quality cells (Supp Fig. 3), and performed unsupervised clustering using the Louvain algorithm [31, 61]. We further refined clustering using the Single-Cell Clustering Assessment Framework (SCCAF), which iteratively applies a machine learning approach to define a cell identity with high accuracy [35]. One bioinformatic feature, resolution, impacts the number of clusters. To determine an ideal resolution, we used Clustree [33] which determines stable clusters at diverse resolution values (Supp Fig. 4a). We constructed four sequencing libraries of control and cuprizone-fed mice from two independent experiments with minimal batch effects (Supp Fig. 4b). Overall, we obtained 10,041 cells, which were reduced to 9225 after quality control. To ensure an equal balance from each library, we down-sampled all libraries to obtain 1000 cells/library, or 4000 total cells. We projected cells onto a Uniform Manifold Approximation and Projection (UMAP) to visualize clustering.

Cell dissociation can cause an artifactual ex vivo activation of microglia characterized by the expression of immediate early and stress-induced genes [62]. We dissociated tissue under cold conditions to prevent ex vivo microglial activation. We found limited expression of both immediate-early genes *Fos* and *Jun* and stress-induced genes *Zfp36* and *Dusp1*, suggesting minimal ex vivo activation (Supp. Figure 4c).

We initially clustered all cells and identified contamination of ependymal cells (Supp Fig. 5a-d) based on the expression of *Calml4*, *S100b*, *Vim*, and *Tmsb10* [63, 64]. Microglia were identified based on the expression of *Hexb, Fcrls, P2ry12,* and *Tmem119* and BAM were defined based on *Lyve1, Ms4a7, *and* Mrc1* [51, 60, 65] (Supp Fig. 5 a-d)*.* We re-clustered microglia and BAMs to obtain 9 clusters: one BAM/macrophage, three cuprizone associated microglia (CAM), three naïve white matter associated microglia (WAM), and 2 clusters containing a mixture from control and cuprizone libraries (Mixed) (Fig. 2b). The BAM cluster also expressed genes linked to monocytes, such as *Ccr2,* suggesting that this cluster may be a mixture of monocytes and BAM (Supp Fig. 5d). The 3 WAM clusters contained cells almost exclusively from control mice and expressed microglia homeostatic markers *Hexb, Fcrls, P2ry12,* and *Tmem119* (Supp Fig. 5b). We found no newly expressed genes that differentiated these different WAM states, but instead, these WAM clusters were derived from more modest differences in gene expression. For example, WAM1 expressed higher levels of several complement protein genes such as *C1qa, C1qb*, and *C1qc,* as well as genes related to cell motility such as *Actb* and *Fcer1g* (Supp Fig. 5e). Therefore, the WAM1 subpopulation is likely primed toward a more motile and synapse pruning phenotype, consistent with the findings of others [56].

Microglia predominate during demyelination and their depletion throughout cuprizone consumption prevents demyelination and preserves myelin ultrastructure [11]. Thus, microglia may cause, or enhance, cuprizone-induced demyelination. Therefore, we aimed to dissect the microglial state associated with the cuprizone diet. We identified three microglial clusters highly enriched in microglia isolated from mice on a cuprizone diet and titled them CAM1, CAM2, and CAM3 (Fig. 2b, c). Consistent with microglial reactivity, CAM subpopulations expressed high levels of lipid regulating genes such as the cholesterol metabolism gene *Apoe* and the lipid catabolic enzyme lipoprotein lipase (*Lpl*) (Fig. 2d), both of which are commonly elevated within reactive microglia [66, 67]. CAM upregulated the pattern recognition receptor *Clec7a*, which encodes Dectin1/Clec7a, a marker of the reactive microglial phenotype common across several neurodegenerative conditions [66, 67]. Indeed, microglial Dectin1 expression was elevated as early as two weeks after mice started the cuprizone diet (Supp Fig. 6. CAM also express *Axl,* a Tyro3-Axl-Mertk receptor tyrosine kinase subfamily member (Fig. 2e). This family of receptors promotes phagocytic clearance of apoptotic cells based on the exposure of the ‘eat-me’ signal, phosphatidylserine [68]. Taken together, CAM subpopulations likely have enhanced phagocytic capacity. CAM also expressed the chemokine *Ccl3*, a factor that accelerates demyelination [69] and *Fth1*, a component of the iron storage protein Ferritin (Fig. 2e). Iron is enriched within oligodendrocytes [70] and the heightened Fth1 may represent an iron accumulation in microglia following oligodendrocyte death and subsequent clearance by microglia. We clustered the differentially expressed genes using hierarchical cluster analysis to determine a gene signature for each identified microglial subpopulation. We obtained four unique gene sets based on 227 genes. The WAM were primarily restricted to gene sets one and two and the CAM to sets three and four (Fig. 2f, Supp Table 1).

**Fig. 3:**
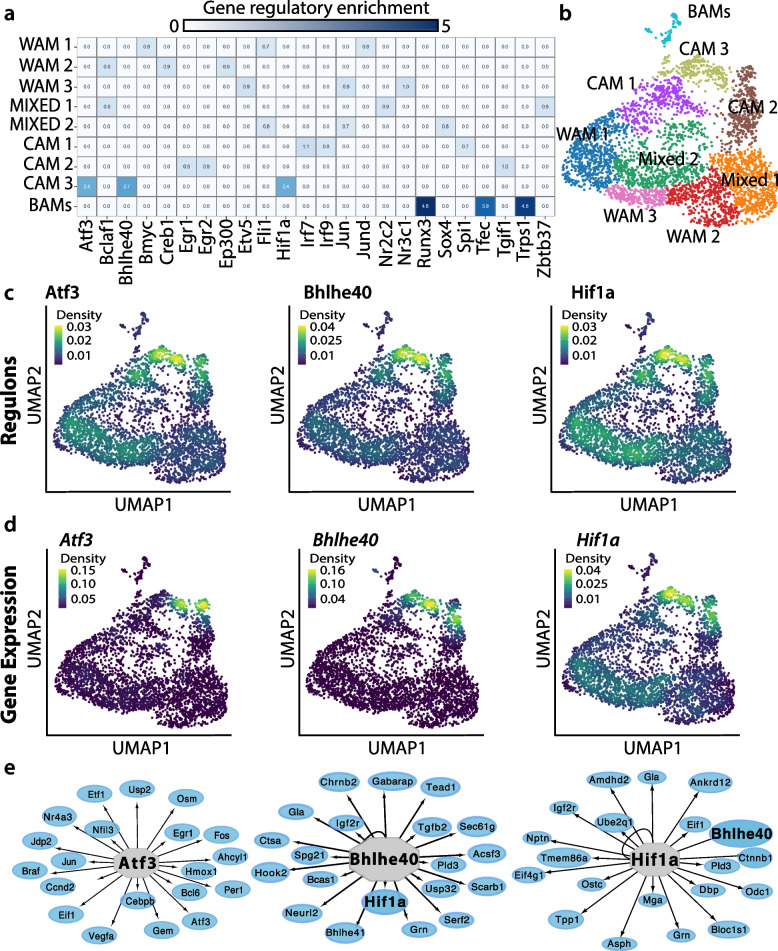
Gene regulatory networks (GRN) of microglia demonstrate cuprizone induces a distinct state.** a** GRN of microglia and BAM clusters (**b**) from corpus callosum microglia isolated from naïve mice and mice fed cuprizone for five weeks. Legend represents the regulon specificity score with blue indicating higher Z-score values i.e., higher expression. **c** Unsupervised graph-based clustering onto a UMAP of SCENIC derived GRN scores and (**d**) gene expression from single cell RNA sequencing dataset highlighting three most enriched cuprizone associated microglia regulons: Atf3, Bhlhe40, and Hif1α. **e** Genes identified by SCENIC as being regulated by Atf3, Bhlhe40, and Hif1α

Given that transcription factors and their associated networks regulate gene expression, we reasoned that more distinctive microglial states are likely to have unique gene regulatory networks. To examine microglial regulation, we conducted single-cell regulatory network interference and clustering (SCENIC) analysis [71]. This tool assesses the gene regulatory networks (GRN)—a set of transcription factors, regulators, and downstream targets—of defined cell clusters to understand how microglial clusters are transcriptionally regulated. SCENIC measures transcription factors and co-regulated genes with significant motif enrichment for a given transcription factor. We found the BAM cluster to be strongly enriched for three regulons linked to macrophages: *Runx3*, *Tfec*, and *Trsp1* (Fig. 3a). Runx3 regulates TGFβ related genes within colonic mononuclear phagocytes [72], while Tfec is a macrophage-specific transcription factor important in regulating several IL4-response genes [73, 74].

The CAM3 cluster was also distinct, containing three enriched regulons: Atf3, Bhlhe40, and Hif1α (Fig. 3a–d). The roles of these transcription factors are unknown during cuprizone demyelination. Atf3 is stimulated by endoplasmic reticulum stress, cytokines, chemokines, and lipopolysaccharide (LPS), suggesting it is a hub for different forms of cellular stress and inflammatory signals [75]. Using the Cytoscape  iRegulon plugin, we identified Atf3 as an essential regulator of several immediate early genes, such as *Fos, Jun*, and *Egr1* (Fig. 3e)*,* that are related to a more surveillant microglial state [76]. Interestingly, Bhlhe40 and Hif1α regulate each other, suggesting they are part of an interconnected transcriptional network (Fig. 3e). Little is known about Bhlhe40 in microglia, but Hif1α protein is regulated by oxygen levels and its transcriptional activity is enhanced during hypoxia [77]. Heightened Hif1α is surprising because the cuprizone diet does not induce hypoxia [78, 79]. However, *Hif1a* mRNA also regulates cytokine secretion [80]. *Hif1a* acts as a metabolic regulator known to enhance glycolysis and upregulate glucose transporters [81]. Consistent with heightened microglial Hif1α, CAM express heightened glycolysis genes pyruvate kinase isozyme M2 (*Pkm2*), lactate dehydrogenase (*Ldha*), and Glut6 (*Slc2a6*), a glucose transporter enriched in macrophage stimulated with the LPS [82] (Supp Fig. 7a). We examined the genes that Hif1α and Bhlhe40 were predicted to regulate (Fig. 3e) in CAM states and found CAM subpopulations upregulated genes involved in cellular metabolism (*Gla, Ctsa, Asph*) and lysosomal function (*Pld3, Gabarap, Grn, Gla*). Therefore, cuprizone induces a distinct microglial state enriched with Atf3, Bhlehe40, and Hif1α gene regulatory networks resulting in a shift towards a more inflammatory state characterized by greater glycolysis and altered lysosomal function.

**Fig. 4:**
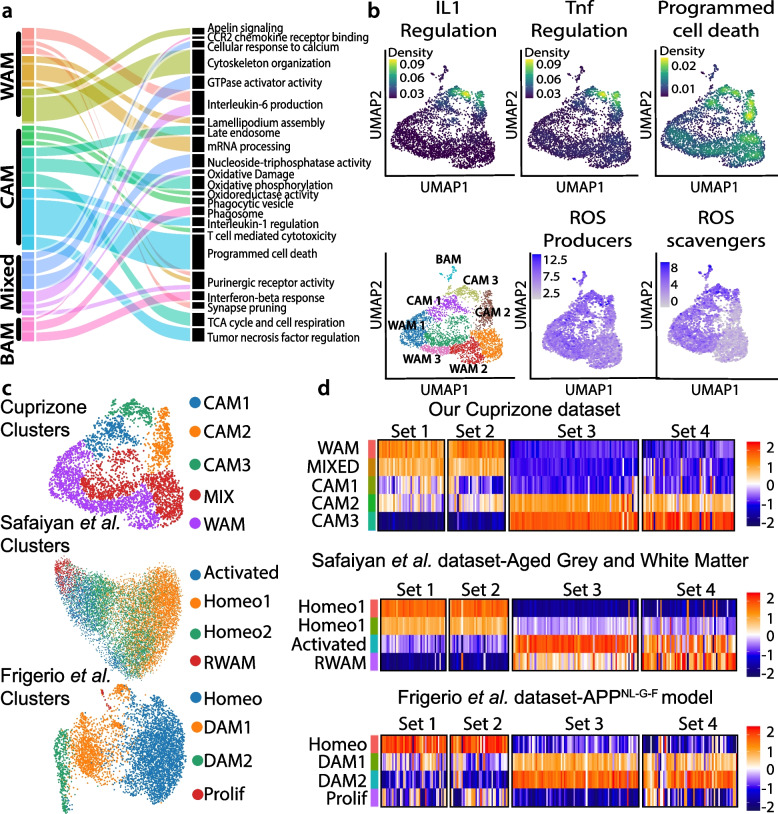
Cuprizone-associated microglia are linked to inflammatory processes and reactive oxygen species production and resemble “disease associated microglia”. **a** Alluvial plot linking microglial clusters from naïve and five weeks post-cuprizone dataset to gene ontology terms. **b** Gene ontology terms projected onto UMAP of naïve and five weeks post-cuprizone microglia. ROS producer and ROS scavenger genes were compiled, summed, and projected onto a UMAP projection. **c** Unsupervised graph-based clustering onto a UMAP for cuprizone dataset combining clusters identifies five clusters: cuprizone associated microglia (CAM) 1 to 3, mixed, and white matter associated microglia (WAM). Safaiyan et al. 24 month old grey and white matter dataset contained four clusters: homeostatic (homeo), reactive white matter microglial (RWAM), and activated [55]. Frigerio et al. dataset with 3, 6, 12, and 21 month-old APP^NL-G-F^ mice contained four clusters: disease associated microglia (DAM)1, DAM2, proliferative microglia (Prolif), and homeo [56]. **d** Cuprizone dataset, Safaiyan et al. dataset, and Frigerio et al. dataset were compared using the four gene sets derived from the differentially expressed genes from cuprizone dataset (this study). Data was normalized and red represents higher average expression while blue represents lower average expression

To understand potential CAM functions, we synthesized microglial gene expression according to gene ontology (GO) annotations. Here, we compared each cluster’s top 50 differentially expressed genes to the functional pathways available in the gene ontology (molecular function, cellular component, and biological processes), KEGG and REACTOME databases to identify enriched processes within different clusters. From this, we determined that CAM expressed genes related to phagocytosis (phagocytic vesicle and late endosome), metabolism (oxidative phosphorylation, oxidoreductase activity, and cell respiration), and cytokine regulation (interleukin-1 (IL1) regulation and tumor necrosis factor (TNF) regulation) (Fig. 4a, b). As a phagocytic microglia state is linked to increased oxidative phosphorylation and cellular respiration in development [83], these metabolic pathways may indicate a highly phagocytic CAM state. The average gene expression for GO annotations were projected onto the microglia clusters demonstrating that CAM 2 and CAM 3 upregulate genes associated with programmed cell death, IL1 regulation, and TNF regulation.

Microglia and astrocytes signal to each other [84], which may be important in determining the CAM state. We used a bioinformatic tool, called NicheNet [36], to gain insight into potential astrocyte ligands that may regulate the CAM state. We compared homeostatic or immune-boosted astrocyte datasets [37] with our CAM single cell transcriptomics (Supp Fig. 8). We found homeostatic astrocytic factors such as *Apoe* and *Fgf1* were potential regulators of the CAM state. By contrast, astrocytes from animals treated with the immune stimulant lipopolysaccharide (LPS) were enriched with *Bmp7, Fgf1, Apoe*, and *Csf1* which were potential regulators of the CAM state.

Given that inflammatory conditions can enhance reactive oxygen species (ROS) production in microglia [85], we used KEGG and REACTOME databases and literature sources to identify ROS-producing and -scavenging enzymes (Supp. Table 2). We summed and projected ROS enzymes onto microglial UMAPs and found that CAM subpopulations expressed higher levels of ROS-producing and -scavenging enzymes (Fig. 4b, Supp Fig. 7b). Despite this upregulation, we found the CAM state was associated with heightened lipid peroxidation as defined by the deposition of malonaldehyde within the corpus callosum after four weeks on a cuprizone diet (Supp Fig. 7c). Therefore, cuprizone results in a microglial state associated with proinflammatory cytokine regulation, phagocytosis, and ROS production.

To understand the transitioning of microglial subpopulations between WAM and CAM states, we used scVelo to calculate RNA velocity, which leverages each cell’s spliced and unspliced information to determine the rates of active transcription and repression [38]. We further explored the data using CellRank, an algorithm that models cell state dynamics to identify initial and terminal cell states based on the RNA velocity data [39]. To our surprise, CellRank identified CAM3 as the initial cell state with terminal conditions in WAM1, WAM2, and WAM3, potentially suggesting that the CAM state was directed toward the resolution of inflammation (Supp Fig. 9a-d). We observed multiple trajectories between homeostatic and the CAM3 subpopulation, with terminal states residing within several WAM subpopulations and a single initial state in CAM3 (Supp Fig. 9c). We identified multiple cellular trajectories between CAM3 towards WAM1, WAM2, and WAM3, suggesting CAM3 is a convergent state between multiple microglial homeostatic clusters (Supp Fig. 9c). To understand the genes that drive the transition into each subpopulation, we examined the genes with dynamic velocity across latent time (Supp Fig. 9e). We identified *Apoe* and *Apoc1* as dynamically regulated genes as they both contain cells with more unspliced transcripts, indicative of increased transcription of these genes in CAM3 cells (Supp Fig. 9f). From this work, we identify CAM3 as a coalescent cellular state.

Currently, microglial states are defined for several different ages and conditions, including during development [54, 86–88], lysophosphatidylcholine (LPC)-induced demyelination [50, 54], within amyloid producing Alzheimer’s disease models or brains [56, 67, 89], during aging [55, 90], and within MS and autoimmune models [58, 91, 92]. We compared our data to other published microglial states to understand how CAM relate to microglia from other conditions. We chose available datasets with comparable murine microglial isolations because tissue dissociation methods can impact the microglial state [62]. Safaiyan and colleagues identified a reactive subpopulation of microglia within white matter regions that are more prominent during aging, which we refer to as reactive white matter-associated microglia (RWAM) [55]. Frigerio and colleagues isolated microglia from amyloid-producing APP^NL-G-F^ mice and identified disease-associated microglia (DAM) subpopulations [56, 67]. Based on our gene expression profile, we postulated that the CAM state may be similar to either the RWAM or DAM states. We first merged our homeostatic WAM and mixed clusters to compare the microglial states from our datasets with others (Fig. 4c). We then clustered microglia from 24-month grey and white matter [55] and 3, 6, 12, 21 month-old APP^NL-G-F^ mice [56] (Fig. 4c). From aged white matter we confirmed the original clustering to identify RWAM and activated microglial subpopulations [55]. The activated microglial subpopulation was enriched with genes responsible for translation, like those in our gene set 3 (Fig. 4d). We compared the gene signature of the CAM, RWAM, and DAM states using the identified CAM-based gene sets (Fig. 2f). We found that CAM2 and CAM3 most closely resembled DAM1 and DAM2 states from APP^NL-G-F^ mice (Fig. 4d), similar to the findings of others [93]. Overall, we find CAM subpopulations resemble the transcriptional state of microglia associated with amyloid plaques more closely than those in the aging white matter.

### Microglia consume lytic carcasses

**Fig. 5:**
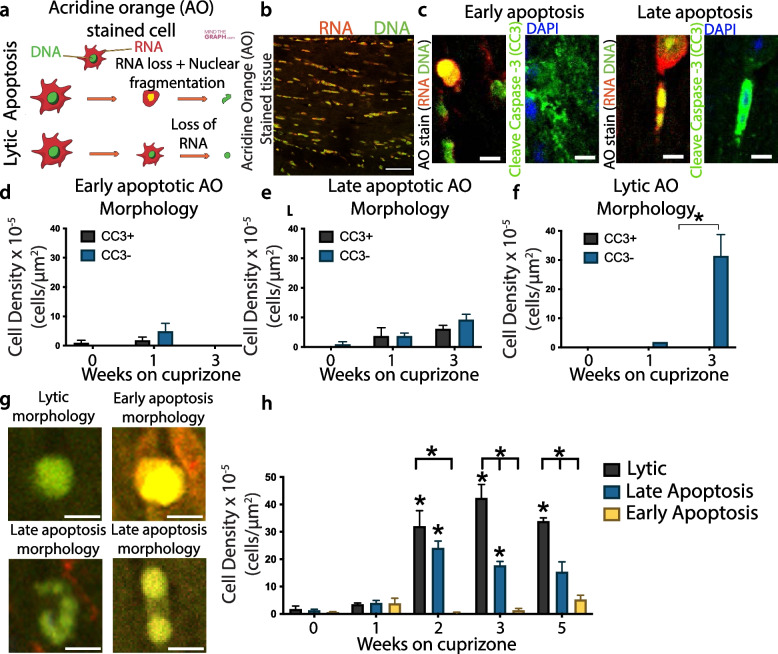
Cuprizone induces diverse forms of cell death. **a** Schematic of acridine orange (AO) fluorescence for lytic and apoptotic cells from [97]. **b** Representative images from AO-stained tissue highlighting green nuclear (DNA) and red cytoplasmic (RNA) fluorescence. (**c**) Representative AO images of ‘early apoptosis’ and ‘late apoptosis’ morphologies based on red condensing and fragmented morphologies, respectively. AO images were matched with comparable immunohistograph images with the apoptotic marker cleaved caspase 3 (CC3). (**d**) Early apoptotic, (**e**) late apoptotic, and (**f**) lytic cells stratified by CC3 expression were quantified from control animals and those on a cuprizone diet. **g** Representative morphologies of early apoptotic, late apoptotic, and lytic cells based on AO fluorescence. **h** Quantification of cell death based on AO fluorescence across a five-week cuprizone time course. (**d-f**) *n* = 2–3 (**h**) *n* = 3–7; Two-way ANOVA with Tukey’s multiple comparisons test (*p* < 0.05). Error bar ± SEM. Scale bar, (**b**) 50 μm (**c, g**) 10 μm

Given the similarities between CAM and DAM states, we asked whether microglia during the cuprizone diet have similar roles to microglia during excess amyloid production. Microglia surround Aβ plaques and phagocytose amyloid, likely to limit damage to neural cells [94, 95]. We postulated that the CAM state may serve a similar role by phagocytosing myelin debris and dead cells during cuprizone-induced demyelination. Cell death occurs through diverse mechanisms with various perturbations of the intracellular or extracellular environments that transduce signals to cause cell death, including different cell death subroutines with lytic (also called necrotic) or apoptotic morphologies [96]. We first wanted to identify the distribution of lytic and apoptotic morphologies during cuprizone. Cuprizone diet induces diverse mechanisms causing oligodendrocyte cell death, including both apoptotic and lytic forms such as ferroptosis, necroptosis, and pyroptosis [22]. We previously found that loss of cellular RNA occurs due to diverse cell death mechanisms [97]. RNA and DNA can be visualized by the nucleic acid dye acridine orange (AO) and used to differentiate morphological attributes indicative of lytic and apoptotic cell death. AO stains DNA with green fluorescence (490-550 nm wavelength) and RNA with red fluorescence (580-750 nm wavelength) using 488 nm excitation (Fig. 5a, b).

As a first approach to understand whether microglia clear lytic and apoptotic cells, we first wanted to clarify and validate the use of AO to morphologically distinguish apoptotic and lytic carcasses. We examined cells with abnormal morphology based on AO staining and quantified whether these cells were apoptotic based on the expression of cleaved caspase-3 (CC3). We identified two distinct morphologies of CC3-positive cells: cells with a bright nuclear red staining we termed ‘early apoptosis’ and cells characterized by nuclear fragmentation or two-beaded nuclei we termed ‘late apoptosis’ (Fig. 5c; Supp Fig. 10). In contrast, ‘lytic’ cell death was defined by a lack of cellular RNA and a round, unfragmented nucleus (Fig. 5a, g), which we previously found occurred in conjunction with cell membrane permeability in culture and tissue [97]. We found lytic cells co-labelled with NINJ1 (Supp Fig. 10), a protein responsible for plasma membrane rupture [98, 99]. We then quantified cells with these early apoptotic, late apoptotic, and lytic morphologies along the time course of cuprizone toxicity. Male mice were fed a cuprizone diet for one or three weeks (Fig. 5d–f), stained with AO, imaged, then immunolabelled with CC3 before imaging again. Overall, we found both early and late apoptotic morphologies, but not lytic morphologies, co-labelled with CC3, further verifying that this morphology distinguishes apoptotic from lytic cell death. We then examined cell death with AO before cuprizone-induced demyelination at one and three weeks and after demyelination at five weeks (Fig. 5h). Cell death peaked by week three of cuprizone, with surprisingly high levels of lytic cell death before and after demyelination. The early cell death at two weeks post-cuprizone preceded the microglial expansion between two to four weeks on cuprizone (Fig. 1). Therefore, AO-based morphology can define lytic and apoptotic cell death morphologies, which are found before robust microglial expansion and demyelination.

**Fig. 6:**
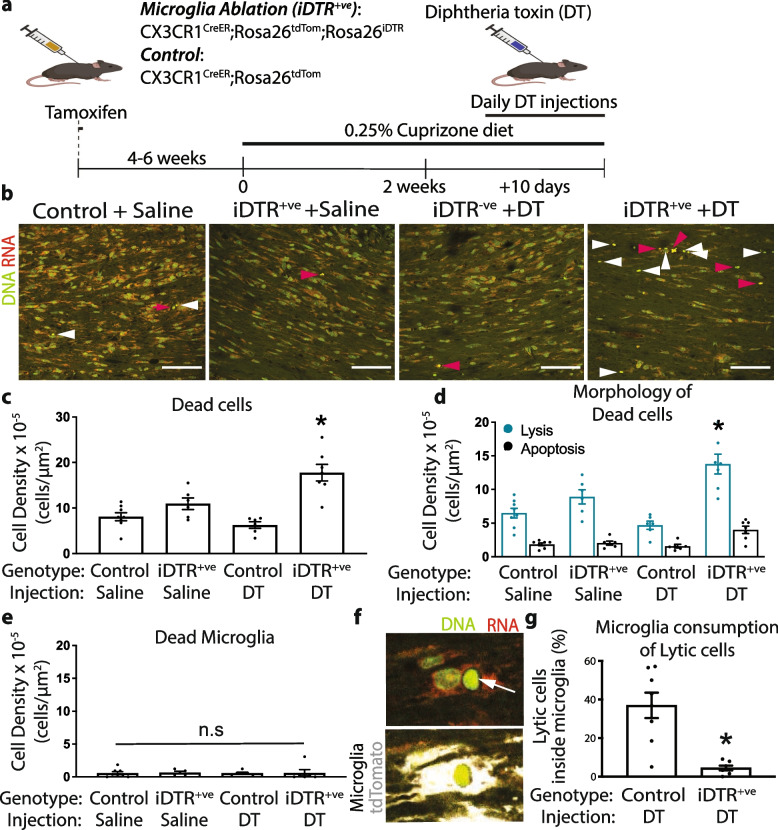
Microglia ablation causes accumulation of lytic carcasses.** a** Schematic of microglia ablation starting two weeks after mice began a cuprizone diet. **b**. Representative corpus callosum acridine orange (AO) stained section imaged after 10 days of diphtheria toxin treatment. Microglia ablation elevated lytic morphologies, based on morphological criteria to differentiate apoptotic morphologies (red arrowhead) and lytic morphologies (white arrowhead). This observation is reflected in quantification of total dead cell density (**c**) and cell density stratified by apoptotic and lytic morphologies (**d**). (**e**) Ten days of diphtheria toxin injections to ablate microglia did not increase the density of dead tdTomato-positive cells (microglia). **f** Representative AO-stained image overlaid with immunohistochemical image demonstrating lytic carcass inside of microglia. **g** In control mice fed cuprizone lytic carcasses were commonly found within microglia, but after microglia ablation, this phenomena was rare. (**c, d, e, f**) *n* = 5–6/group. (**c, e**) One-way ANOVA with Tukey’s multiple comparison test. **d** Two-way ANOVA with Holm-Sidak’s multiple comparisons test. **g** Welch’s T test. Error bar ± SEM. Scale bar, (**b**) 50 μm (**f**) 10 μm

It is still unclear which cells are responsible for clearing dead cells during cuprizone-induced demyelination. To understand the contribution of microglia in the clearance of cell carcasses, we ablated microglia using CX3CR1^creER^; Rosa26^tdTom^; Rosa26^iDTR^ transgenic mice (Fig. 6). These mice insert an inducible diphtheria toxin receptor (iDTR) into CX3CR1 expressing cells following tamoxifen injections. We injected mice with tamoxifen for three days starting at postnatal days 12 or 13 and provided cuprizone four to six weeks later to ensure iDTR is expressed solely within resident macrophages, like microglia. Thus, these mice express tdTomato and iDTR following tamoxifen treatment, allowing us to fate-map and specifically ablate microglia and BAMs via diphtheria toxin (DT) injection [48, 50]. We fed cuprizone for two weeks to induce cell death before injecting diphtheria toxin or saline for ten consecutive days while keeping mice on cuprizone (Fig. 6a). DT injections of iDTR-positive mice significantly lowered the density of microglia in the corpus callosum by 93% compared to iDTR-negative mice (Supp Fig. 11a, b). Mice that were iDTR-positive and treated with saline had no reduction in microglia density, demonstrating a lack of endogenous iDTR activity without DT injection (Supp Fig. 11a, b). Microglia ablation did not alter oligodendrocyte lineage cell density based on Olig2 labelling—a pan-oligodendrocyte lineage marker—suggesting that microglia were not killing oligodendrocyte lineage cells (Supp. Fig. 11c, d). Surprisingly, unlike microglia ablation throughout dietary cuprizone [11], ablation of microglia two weeks after the start of dietary cuprizone did not alter demyelination based on the lipid stain euriochrome cyanine (Supp Fig. 11e, f). Therefore, microglia involvement in demyelination may occur within the first two weeks after the start of dietary cuprizone.

To understand whether microglia ablation changed the clearance of dead cells, we treated CX3CR1^creER^; Rosa26^tdTom^; Rosa^iDTR^ and control mice with cuprizone and ablated microglia starting at two weeks. We then examined AO-stained corpus callosum sections (Fig. 6b). We found that microglia ablation increased the density of dead cells, with an accumulation of lytic, and not apoptotic, carcasses (Fig. 6c, d). Importantly, we did not observe a rise in dead microglia—based on tdTomato expression in AO-stained cells with a morphology indicative of death—suggesting that the accumulation of lytic cells was not a consequence of microglia ablation (Fig. 6e). We conclude that microglia were responsible for clearing lytic carcasses. Indeed, we identified ~ 35% of lytic carcasses inside tdTomato-positive microglia, which dropped to ~ 4% after microglia ablation (Fig. 6f, g). Given that apoptosis was ongoing during cuprizone consumption (Fig. 5h) and that apoptotic carcasses did not accumulate after microglial ablation, we reason other phagocytic glial cells support the clearance of apoptotic corpses. From this work, we conclude that apoptotic carcasses may be cleared by several glial cells, while microglia are the major contributor to the clearance of lytic cell carcasses.

### Microglia exposure to dead oligodendrocyte components partially recapitulates CAM state

**Fig. 7:**
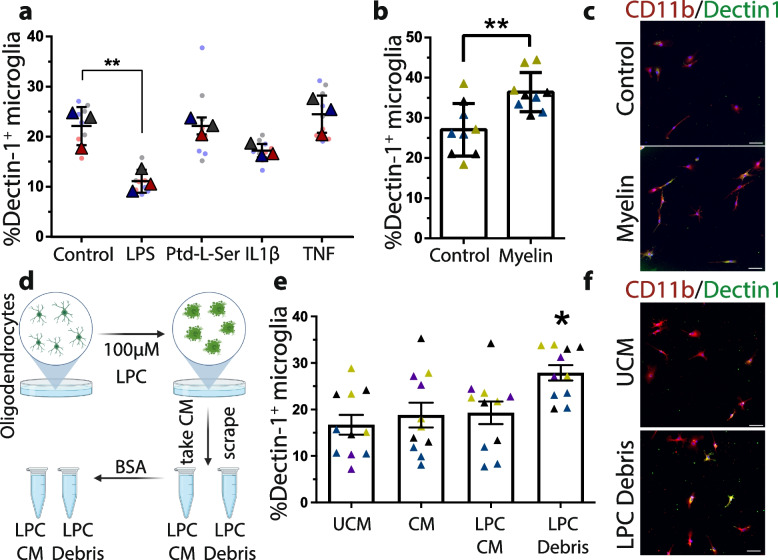
Exposure to myelin and dead cellular debris stimulates CAM marker Dectin1/Clec7a in microglial culture. **a** Using purified, serum-free, microglial cultures, proinflammatory cytokines and immune stimulants failed to increase the proportion of microglia expressing Dectin1. **b, c** Quantification and representative immunocytochemical image demonstrating myelin debris exposure increases the proportion of Dectin1 expressing microglia, in purified, serum-free, cultures. **d** Schematic illustrating experimental procedure to kill cultured oligodendrocytes with LPC and subsequent chelation of LPC with albumin to halt toxicity. **e, f** Quantification and representative immunocytochemical image demonstrating that exposure to LPC-treated oligodendrocyte carcasses stimulates microglial Dectin1. **a, b, e** *n* = 3 wells in 2–3 independent experiments (**a, e**) One-way ANOVA with Dunnett’s multiple comparison test. **b** Unpaired T-Test. Error bar ± SEM. Scale bar, (**c, f**) 50 μm

Knowing that microglia are critical for the clearance of dead cells during cuprizone-induced demyelination and that they form a distinct microglial state, we wanted to understand whether exposure to factors enriched during cuprizone is responsible for their state. To examine how microglia change in culture, we cultured microglia in serum-free conditions as they are more similar to homeostatic microglia than in traditional serum-based cultures [45]. To assess the CAM state, we measured Clec7a/Dectin1 levels in cultured microglia because *Clec7a* defines the CAM state (Fig. 2) but is also increased in response to diverse disease states, including animal models of Alzheimer’s disease, multiple sclerosis, amyotrophic lateral sclerosis, and aging [66]. We first treated microglia with an array of pro-inflammatory factors, including LPS, IL1β, phosphatidylserine, and TNF (Fig. 7a). Surprisingly, these factors did not increase Dectin1 expression in serum-free microglia cultures. Indeed, the classical immune stimulant LPS consistently reduced Dectin1 in microglia. We then postulated that internalization of debris may be required to increase Dectin1. Serum-free microglia do not readily phagocytose myelin debris unless in the presence of serum [45] (Supp Fig. 12a). Exposing cells to pHrodo™ labelled myelin, which fluoresces inside of acidic cellular compartments after phagocytosis and verifying uptake with immunocytochemistry towards myelin basic protein, demonstrates that roughly 10% of microglia can phagocytose myelin debris by 72 hr. even in the absence of serum (Supp Fig. 12a–c). This limited myelin phagocytosis was associated with more microglia that expressed Dectin1 (Fig. 7b, c). Exposure to serum increased the proportion of microglia that phagocytose myelin, which resulted in heightened Dectin1 expression (Supp Fig. 12d–f).

One key component of lytic carcasses is the nucleus. To determine whether microglia can phagocytose nuclei, we isolated pure nuclei and labelled them with pHrodo™. Like myelin, microglia in serum-free conditions do not phagocytose nuclei but phagocytose nuclei in the presence of serum (Supp Fig. 12 g). The addition of nuclei in the presence or absence of serum did not increase Dectin1 in microglial cultures (Supp Fig. 12 h, i). As dietary cuprizone kills myelinating oligodendrocytes [22], we aimed to model lytic carcasses following cuprizone by inducing lytic cell death of oligodendrocytes with the lipid disrupting agent LPC [100]. We treated cultured oligodendrocytes with LPC, then albumin, which chelates LPC and prevents further toxicity [100]. We collected both the conditioned media (CM) and dead cell debris of LPC and albumin-treated oligodendrocytes (Fig. 7d). We found that the CM of dead oligodendrocytes did not increase microglial Dectin1, suggesting that damage-associated molecular patterns do not change Dectin1 in cultured microglia (Fig. 7e). By contrast, the LPC-treated cellular debris, or oligodendrocyte lytic carcasses, significantly increased the proportion of Dectin-1-positive microglia, suggesting some aspect of the dying carcass, and not the lytic nuclei, were required to induce Dectin1 expression in cultured microglia (Fig. 7e, f). We conclude that microglia can phagocytose nuclei present after lytic cell death, but only after treatment with myelin debris or lytic carcasses do they take on a Dectin1-expressing microglial state.

## Discussion

The pathogenesis of cuprizone-induced demyelination is still unclear but likely models aspects of MS pathophysiology. Here, we found that microglia predominate during the progression of cuprizone-induced demyelination, with a minor infiltration of T cells and monocyte-derived macrophages. At peak demyelination, we identified a distinct microglial state characterized by genes involved with pro-inflammatory cytokine regulation and oxidative stress with altered metabolic and lysosomal gene expression. In comparing this microglial state with other disease conditions, we and others [93] find the CAM states highly resemble the plaque-associated DAM states present in other neurodegenerative disorders. Cuprizone induced both apoptotic and lytic forms of cell death weeks before demyelination was present, suggesting that oligodendrocyte injury precedes myelin disruption. We found that lytic carcasses were prominent and that microglia were necessary for their clearance. In culture, the uptake of lytic carcasses recapitulated the expression of Dectin1, a central marker of the CAM state and a microglial neurodegenerative phenotype [66].

The presence of lytic carcasses and myelin debris upregulate elements of the CAM state, suggesting the internalization of cellular components drives this state. Indeed, the related microglia neurodegenerative phenotype is recapitulated by injecting apoptotic cells into the brain [66]. We clarify these findings to show that myelin and dead cells are sufficient to drive this microglial state change within highly pure microglial cultures. What initiates microglia phagocytosis? We found, as did Bohlen and colleagues [45], that serum-free microglia cultures are largely non-phagocytic, suggesting that microglia require a stimulus to phagocytose extracellular debris.

What extracellular elements promote microglial phagocytosis of debris? Myelin debris is laden with phosphatidylserine, which is necessary for its phagocytosis [55]. However, this common ‘eat me’ signal is surprisingly insufficient to drive microglial phagocytosis under basal conditions in serum-free cultures. Antibodies, complement proteins, or fibrin are potential serum-related factors that could push microglia into a phagocytic state. The mild disruption of the blood-brain barrier after cuprizone consumption may be sufficient to encourage microglial phagocytosis [101, 102]. Another factor that could alter microglial phagocytosis is type I interferons, such as interferon (IFN)-β. IFN-β promotes microglial phagocytosis [103, 104] and expression of IFN response elements such as *Irf7*, *Ifitm3*, and *Ifit3* are present in CAM subpopulations suggesting that microglia may respond to type I IFN during cuprizone toxicity. Indeed, the loss of IFN-β during dietary cuprizone results in fewer reactive microglia and more apoptotic carcasses, which is suggestive of a defect in clearance [105]. IFN-β may, therefore, be an essential trigger to enhance phagocytosis resulting in crucial elements of the CAM state.

After microglia phagocytose dead cells, it remains unclear what drives the altered microglial states. Recently Dolan and colleagues exposed human stem cell-derived microglia to synaptosomes, myelin debris, apoptotic neurons, or synthetic amyloid-beta and found that all of these substrates drive a DAM state in culture [106]. Given the high overlap between the CAM and DAM states, microglial phagocytosis is likely a common mechanism causing these disease-associated states. How phagocytosis is linked to DAM states remains an open question but is likely important given the extensive overlap between these states across neurological diseases.

It is well established that microglia can phagocytose apoptotic cells [107]. Astrocytes can also phagocytose synapses [26] and dead cells [27, 28], with astrocytes as a likely cell to compensate for ablated microglia [28]. Alternatively, oligodendrocyte progenitors can also conduct phagocytosis [24, 25] and may also support phagocytosis of carcasses after microglial ablation. While mechanisms for non-microglial phagocytosis are still to be discovered within the CNS, one way astrocytes phagocytose is via the phagocytic receptors Megf10 and Mertk [26], which sense ‘eat me’ signals such as phosphatidylserine [108]. Phosphatidylserine in healthy cells is restricted to the inner leaflet of the lipid bilayer by ATP-dependent translocases [109]. During apoptosis, caspase 3/7 targets the scramblase Xk-related protein 8 (Xrk8) which rapidly flips phosphatidylserine to the outer lipid bilayer leaflet [110]. This phosphatidylserine translocation allows apoptotic cells to be recognized by several phosphatidylserine receptor systems—including those expressed by astrocytes—to facilitate apoptotic cell clearance [108]. By contrast, lytic (also called necrotic) forms of death do not necessarily promote phosphatidylserine translocation to levels allowing cells to be recognized by phagocytes [111]. For this reason, there are alternative mechanisms to tag and promote the phagocytosis of lytic cells, but the mechanisms of this within the CNS remain to be uncovered.

How does the prominent microglial accumulation relate to blood brain barrier (BBB) permeability during dietary cuprizone? The disruption of the BBB is seen as early as three days after cuprizone, based on Dextran or Evans Blue tracer leakage into the brain [101, 102]. Consistent with BBB disruption, tight junction proteins are downregulated by  five-to-seven days post-cuprizone and are further decreased at five weeks post-cuprizone, suggesting BBB disruption for the entire course of cuprizone treatment [101]. This leaky BBB might present points of entry for peripheral immune cells or other immunogenic factors into the brain. However, we found limited entry of T-cells and that the vast majority of myeloid cells in the corpus callosum were microglia. The robust expansion of microglia, with limited monocyte contribution, similarly occurs after facial nerve injury in the facial nucleus [112]. Likewise, limited monocyte recruitment occurs after LPC-induced demyelination, but microglia preferentially expand to monopolize the demyelinated lesion and prevent the movement of monocytes within spared white matter [50]. Unlike the CNS, in the peripheral nervous system monocytes are the major source of phagocytes after LPC-induced demyelination. Our current work and these studies highlight that even under inflammatory conditions, monocyte access to, and movement, within the CNS is restricted.

## Conclusions

We identify microglia as the primary immune cell in the corpus callosum during cuprizone-induced demyelination that form a cuprizone-associated microglia (CAM) state. The CAM state is associated with Atf3, Bhlehe40, and Hif1α gene regulatory networks, enriched with genes related to cytokine regulation, and shifts towards a more oxidative phenotype. CAM are required to consume lytic carcasses after cuprizone exposure. The phagocytosis of lytic corpses in culture can partially recapitulate the CAM state, suggesting that clearance alters the microglial phenotype.

## Supplementary Information

**Additional file 1: Supp.** **Fig. 1. **Microglia predominate following cuprizone-induced demyelination.** a** Microglia and BAM express tdTomato (tdTom, red) in CX3CR1^creER^; Rosa26^tdTom^ mice with early tamoxifen injections. Representative immunohistochemical images from CX3CR1^creER^; Rosa26^tdTom^ mice four weeks on a cuprizone diet with border-associated macrophage (BAM) marker (Lyve1, green) and pan-leukocyte marker (CD45, white) depict low BAM presence during cuprizone-induced demyelination. **b** There was a minor T cell presence at four weeks on a cuprizone diet in CX3CR1^creER^;Rosa26^tdTom^ mice. Representative immunohistochemical images with T cell marker (CD4, green) and pan-leukocyte marker (CD45, white) in dorsal (**d**) and ventral (**e**) inlets. (**c**) CD4 was validated in tissue from lymphocyte-driven experimental autoimmune encephalomyelitis (EAE). (**d**) Example of T cell (CD45^+ve^, CD4^+ve^, tdTom^-ve^) in the dorsal inlet, and example of likely monocyte (CD45^-ve^, CD4^+ve^, tdTom-ve) in ventral inlet. (**f, g**) Immunohistochemical images demonstrate neutrophils (Ly6G, green) are absent from the CNS parenchyma, tdTom (red). Ly6G (green) antibody was validated in the EAE MS animal model. Scale bar, (**a, b, c, f, g**) 100 μm, (**d, e**) 50 μm. **Supp.** **Fig. 2.** Microglia proliferate and phagocytose myelin debris during cuprizone-induced demyelination. **a** Representative images of microglia expressing tdTomato (tdTom, red) in CX3CR1^creER^;Rosa26^tdTom^ mice express the proliferative marker (Ki67) at 2 weeks and 4 weeks after the start of dietary cuprizone. **b** Representative images of microglia (tdTomato-positive cells) that contain the major myelin protein, myelin basic protein (MBP). The MBP aggregates inside of microglia was consistent with microglial phagocytosis of myelin debris. Scale bar, (**a)** 40 μm, (b) 20 μm. **Supp.** **Fig. 3.** Single-cell RNA sequencing quality control data. For quality control the proportion of mitochondrial genes and the number of genes were plotted against total number of molecules present in each cell (nCount) in thousands, for each library of control and 5 week cuprizone experiments. **Supp.** **Fig. 4.** Single-cell RNA sequencing approaches and validation. (**a**) A Clustree plot was used to determine optimal resolution. As resolution increases, moving down the clustering tree plot from 0 to 1.0, there is an increase in the number of clusters. With more clusters, there are fewer cells in each cluster (represented by circle size). As clusters split, arrows show the movement of cells from one cluster into multiple new clusters; the proportion of cells is indicated by arrow opacity. We chose resolution (0.5), where the mixing of cells is minimal. (**b**) Unsupervised graph-based clustering of individual libraries used in the larger dataset projected onto a UMAP. Control cells from two independent experiments, Library 1 (blue) and Library 2 (green), were primarily interspersed in the same regions. Likewise, cuprizone cells from Library 1 (orange) were largely interspersed with cuprizone cells from Library 2 (red), suggesting minimal batch effects. (**c**) Immediate early genes (*Jun* and *Fos)* and stress-induced genes (*Zfp36* and *Dusp1*) were not stimulated or concentrated in microglial clusters. Legend represents umi score with yellow representing a higher score. **Supp.** **Fig. 5.** Single-cell RNA sequencing annotation and homeostatic gene expression shift. (**a**) Unsupervised graph-based clustering and machine learning-based reclustering of dataset projected onto a UMAP. Cells were annotated guided by markers for (**b**) microglia, (**c**) Ependymal cells, and (**d**) border-associated macrophages (BAM). (**e**) Homeostatic microglia (control cell) subpopulations were located at the bottom of the microglial UMAP. We identified gene expression of complement protein genes (*C1qa, C1qb, C1qc*) and cell motility (*Actb* and *Fcer1g*) that were enriched within some homeostatic microglia subpopulations (microglia, bottom left) but not others (microglia, bottom right). Density represents the kernel density estimation, which resolves gene expression sparsity and rescues drop-out genes based on neighbouring clusters. **Supp.** **Fig. 6. **Dectin1/Clec7a was enriched within microglia within two weeks after initiating the cuprizone diet. Quantification and representative immunohistochemical images demonstrating microglia (tdTomato) express Dectin1 at two and four weeks after initiating the cuprizone diet. *n* = 3 for control and *n* = 6 for 2 and 4 weeks post. One-way ANOVA with Tukey’s multiple comparison test. Error bar ± SEM. Scale bar, 50 μm. **Supp.** **Fig. 7. **Cuprizone-associated microglia gene transcriptional enrichment in glycolysis and reactive oxygen species (ROS) production. (**a**) Microglia and BAM clusters projected onto a UMAP show enrichment of genes related to glycolysis (*Pkm2, Ldha*) and glucose transport (*Glut6*, or *Slc2a6*). Density represents the kernel density estimation, which resolves gene expression sparsity and rescues drop-out genes based on neighbouring clusters. (**b**) ROS producers and scavengers plotted on a heat map with normalized gene expression derived from cluster identity. (**c**) Fold change with ROS producing and scavenging enzymes. Fold change was calculated with averaged gene expression for [(CAM3-WAM1)/(CAM3 + WAM1)]. (**d**) Quantification and representative immunohistochemical images demonstrating lipid peroxidation (Malonaldehyde, or MDA, white) located within regions associated with microglia (IBA1, red) accumulation at four weeks after initiating cuprizone diet. *n* = 5–7 with One-way ANOVA with Dunnett’s multiple comparison test. Error bar ± SEM. Scale bar, (**d**) 100 μm. **Supp. Fig. 8.** Potential astrocyte factors that regulate the cuprizone associated microglial (CAM) state. We used NicheNet to compare (**a**) homeostatic astrocytes and (**b**) astrocytes from LPS-treated mice from Hasel and colleagues’ datasets [37] with CAM. NicheNet compared known secreted factors from these astrocyte datasets with CAM expressed receptors, but also examined the downstream genes regulated by these receptor systems. NicheNet calculates a regulatory potential for CAM genes based on known receptor-ligand interactions and identified transcriptomic expression levels. Naive astrocyte ligands such as *Apoe* and *Fgf1,* and LPS-stimulated astrocyte ligands like *Bmp7, Fgf1, Apoe*, and *Csf1*were identified as potential regulators of the CAM state. **Supp. Fig. 9. **Through trajectory analysis, cuprizone-associated microglia (CAM) subpopulations are linked to white matter-associated microglia (WAM) subpopulations. Cell Rank was used to identify the (**a**) initial and (**b**) terminal predicted states. (**c**) ScVelo was used to determine the cell path and trajectory of cells proceeding from one subpopulation to another. (**d**) Latent time describes the probability of being at an initial state (0, purple) or terminal state (1, yellow). (**e**) A gene expression heat map shows those genes that increase as cells transition from different states across latent time. Legend represents gene expression, with yellow representing a higher value and blue depicting a lower value. (**f**) CellRank was used to identify potential driver genes or genes involved in a fate choice between different states. We interrogated potential driver genes involved in the transition between CAM3 and other microglial states to identify the genes *Apoe* and *Apoc1.* These genes were plotted such that each dot represented a cell whose color provides cluster identity. The cell is plotted based on the extent of unspliced vs. spliced mRNA to depict RNA velocity. **Supp. Fig. 10** Acridine orange (AO) based cell death morphologies compared to membrane lysis protein Ninjurin-1 (NINJ1) and apoptosis protein product cleaved caspase-3 (CC3). (**a**) Representative images of cell death based on AO morphologies that were lytic, early apoptotic, and late apoptotic and their accompanying expression of NINJ1, a marker of plasma membrane rupture. We found prominent expression of NINJ1 in lytic cells, but limited expression within cells with apoptotic morphologies. (**b**) Representative images of lytic, early apoptotic, and late apoptotic cell populations and the corresponding expression of cleaved caspase-3 (CC3), an apoptosis marker. Cells with apoptotic morphologies often expressed CC3, but cells with lytic morphologies expressed little to no CC3 expression. Scale bars, (a, b) 10 μm. **Supp. Fig. 11. **Microglial ablation two weeks after mice are placed on dietary cuprizone does not alter demyelination. (**a, b**) Representative immunohistochemical images and quantification demonstrating that microglia become ablated in mice expressing inducible DTR mice (CX3CR1^CreER^; Rosa26^iDTR^) treated with diphtheria toxin (DT), but not in control CX3CR1^CreER^ mice treated with saline (Control + Saline), CX3CR1^CreER^ mice treated with DT (Control + DT), or CX3CR1^CreER^; Rosa26^iDTR^ mice treated with saline (DTR^+ve^ + saline). (**a**) The top image was taken with lower primary magnification (10x) compared to the lower image (40x). (**c, d**) Quantification and representative immunohistochemical images showing microglia ablation did not change oligodendrocyte lineage cell density (Olig2, white). (**e, f**) Representative widefield image of euriochrome cyanine and neutral red-stained sections with quantification showing no difference in demyelination between control CX3CR1^CreER^ with DT (control + DT) mice and CX3CR1^CreER^; Rosa26^iDTR^ mice treated with DT (DTR^+ve^ + DT). (B,D,F) *n* = 4–7. (**b, d**) One-way ANOVA, (**f**) Unpaired T-Test. Scale bar, (**a**-Top, **e**) 100 μm, (**a**-bottom) 50 μm, (**c**) 20 μm. **Supp. Fig. 12. **Microglia phagocytosis of myelin and nuclei in a serum-free culture system. (**a**) There was limited phagocytosis of pHrodo labelled myelin by 72 hr. in serum-free microglial cultures. Representative images (**b**) and quantification (**c**) comparing pHrodo labelled myelin with the major myelin protein, myelin basic protein (MBP), following immunocytochemistry (ICC) demonstrates that serum increases myelin phagocytosis. (**d**) Based on pHrodo fluorescence, microglia phagocytosis was inhibited with cytochalasin D (CytoD). Representative immunocytchemical image (**e**) and quantification (**f**) demonstrating myelin debris exposure increases the proportion Dectin1 expressing microglia, in purified, serum-free and serum-containing (FBS) cultures. (**g**) Microglia phagocytose pHrodo labelled nuclei when they are treated with FBS, but not under serum-free conditions or with the phagocytosis inhibitor CytoD. Neither the exposure to nuclei under serum-free (**h**) or FBS (**i**) conditions promoted more expression of the cuprizone-associated microglia marker Dectin1/Clec7a. (**h**) *n* = 3 wells in 2 independent experiments or one (**a, c, f, i**) independent experiment. Error bar ± SEM. Scale bar, (***b, e***) 50 μm.

**Additional file 2. Comparison of cuprizone, Frigerio, and Simmons data sets across common gene sets.**

**Additional file 3. Transcriptomic information of ROS producers and scavengers comparing different microglial states in cuprizone dataset.**

## Data Availability

Data is available through the gene expression omnibus (GSE207750).

## Abbreviations

AO: Acridine orange
BAM: Border-associated macrophages
CAM: Cuprizone-associated microglia
CM: Conditioned media
CNS: Central nervous system
DAM: Disease-associated microglia
DEG: Differentially-expressed genes
FACS: Fluorescence-activated cell sorting
GO: Gene ontology
GRN: Gene regulatory network
iDTR: Inducible diphtheria toxin receptor
LPC: Lysophosphatidylcholine
LPS: Lysophosphatidylserine
MS: Multiple sclerosis
ROS: Reactive oxygen species
RWAM: Reactive white matter-associated microglia
SCCAF: Single-cell clustering assessment framework
pySCENIC: Python-based single-cell regulatory network inference and clustering pipeline
scRNAseq: Single-cell RNA sequencing
UMAP: Uniform manifold approximation and projection
WAM: White matter-associated microglia
