# Supplementary material for: Single-cell microglial transcriptomics during demyelination defines a microglial state required for lytic carcass clearance
Source: Mol Neurodegener. 2022 Dec 13;17:82. doi: 10.1186/s13024-022-00584-2 (PMC9746011; doi:10.1186/s13024-022-00584-2)
Supplement: Supplementary file 1 — Additional file 1: Supp. Fig. 1. Microglia predominate following cuprizone-induced demyelination. a Microglia and BAM express tdTomato (tdTom, red) in CX3CR1creER; Rosa26tdTom mice with early tamoxifen injections. Representative immunohistochemical images from CX3CR1creER; Rosa26tdTom mice four weeks on a cuprizone diet with border-associated macrophage (BAM) marker (Lyve1, green) and pan-leukocyte marker (CD45, white) depict low BAM presence during cuprizone-induced demyelination. b There was a minor T cell presence at four weeks on a cuprizone diet in CX3CR1creER;Rosa26tdTom mice. Representative immunohistochemical images with T cell marker (CD4, green) and pan-leukocyte marker (CD45, white) in dorsal (d) and ventral (e) inlets. (c) CD4 was validated in tissue from lymphocyte-driven experimental autoimmune encephalomyelitis (EAE). (d) Example of T cell (CD45+ve, CD4+ve, tdTom-ve) in the dorsal inlet, and example of likely monocyte (CD45-ve, CD4+ve, tdTom-ve) in ventral inlet. (f, g) Immunohistochemical images demonstrate neutrophils (Ly6G, green) are absent from the CNS parenchyma, tdTom (red). Ly6G (green) antibody was validated in the EAE MS animal model. Scale bar, (a, b, c, f, g) 100 μm, (d, e) 50 μm. Supp. Fig. 2. Microglia proliferate and phagocytose myelin debris during cuprizone-induced demyelination. a Representative images of microglia expressing tdTomato (tdTom, red) in CX3CR1creER;Rosa26tdTom mice express the proliferative marker (Ki67) at 2 weeks and 4 weeks after the start of dietary cuprizone. b Representative images of microglia (tdTomato-positive cells) that contain the major myelin protein, myelin basic protein (MBP). The MBP aggregates inside of microglia was consistent with microglial phagocytosis of myelin debris. Scale bar, (a) 40 μm, (b) 20 μm. Supp. Fig. 3. Single-cell RNA sequencing quality control data. For quality control the proportion of mitochondrial genes and the number of genes were plotted against total number of molecules present [file 13024_2022_584_MOESM1_ESM.pdf]

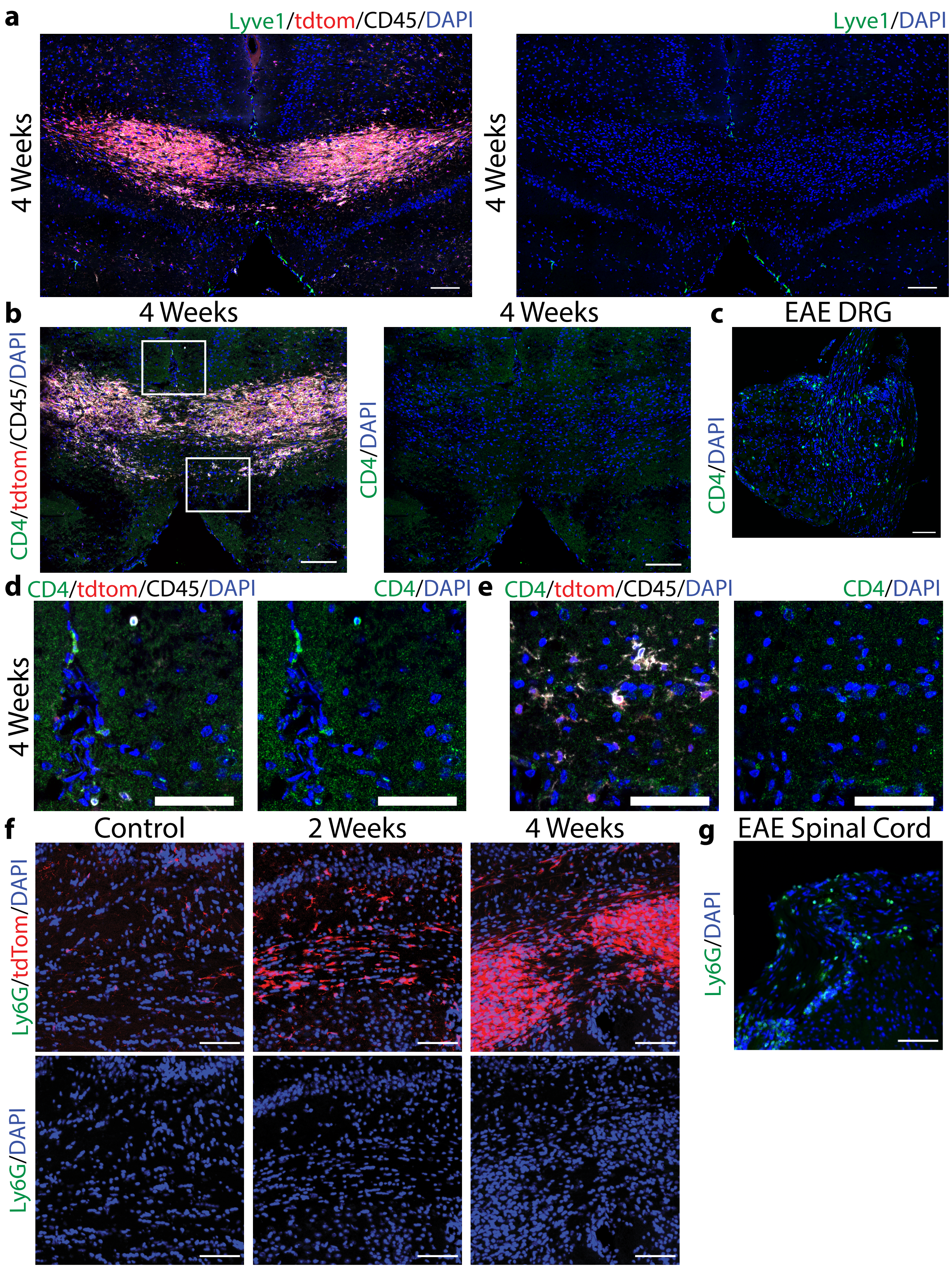

a
Lyve1/tdtom/CD45/DAPI
Lyve1/DAPI
4 Weeks
4 Weeks
b
4 Weeks
4 Weeks
CD4/tdtom/CD45/DAPI
CD4/DAPI
c
EAE DRG
CD4/DAPI
d
CD4/tdtom/CD45/DAPI
CD4/DAPI
4 Weeks
e
CD4/tdtom/CD45/DAPI
CD4/DAPI
f
Control
2 Weeks
4 Weeks
Ly6G/tdTom/DAPI
Ly6G/DAPI
g
EAE Spinal Cord
Ly6G/DAPI

Supp Fig 1. Microglia predominate following cuprizone-induced demyelination. (**a**) Microglia and BAM express tdTomato (tdTom, red) in CX3CR1^{creER}; Rosa^{tdTom} mice with early tamoxifen injections. Representative immunohistochemical images from CX3CR1^{creER}; Rosa^{tdTom} mice four weeks on a cuprizone diet with border-associated macrophage (BAM) marker (Lyve1, green) and pan-leukocyte marker (CD45, white) depict low BAM presence during cuprizone-induced demyelination. (**b**) There was a minor T cell presence at four weeks on a cuprizone diet in CX3CR1^{creER};Rosa^{tdTom} mice. Representative immunohistochemical images with T cell marker (CD4, green) and pan-leukocyte marker (CD45, white) in dorsal (**d**) and ventral (**e**) inlets. (**c**) CD4 was validated in tissue from lymphocyte-driven experimental autoimmune encephalomyelitis (EAE). (**d**) Example of T cell (CD45^{+ve}, CD4^{+ve}, tdTom^{-ve}) in the dorsal inlet, and example of likely monocyte (CD45^{-ve}, CD4^{+ve}, tdTom-ve) in ventral inlet. (**f, g**) Immunohistochemical images demonstrate neutrophils (Ly6G, green) are absent from the CNS parenchyma, tdTom (red). Ly6G (green) antibody was validated in the EAE MS animal model. Scale bar, (**a, b, c, f, g**) 100µm, (**d, e**) 50µm.

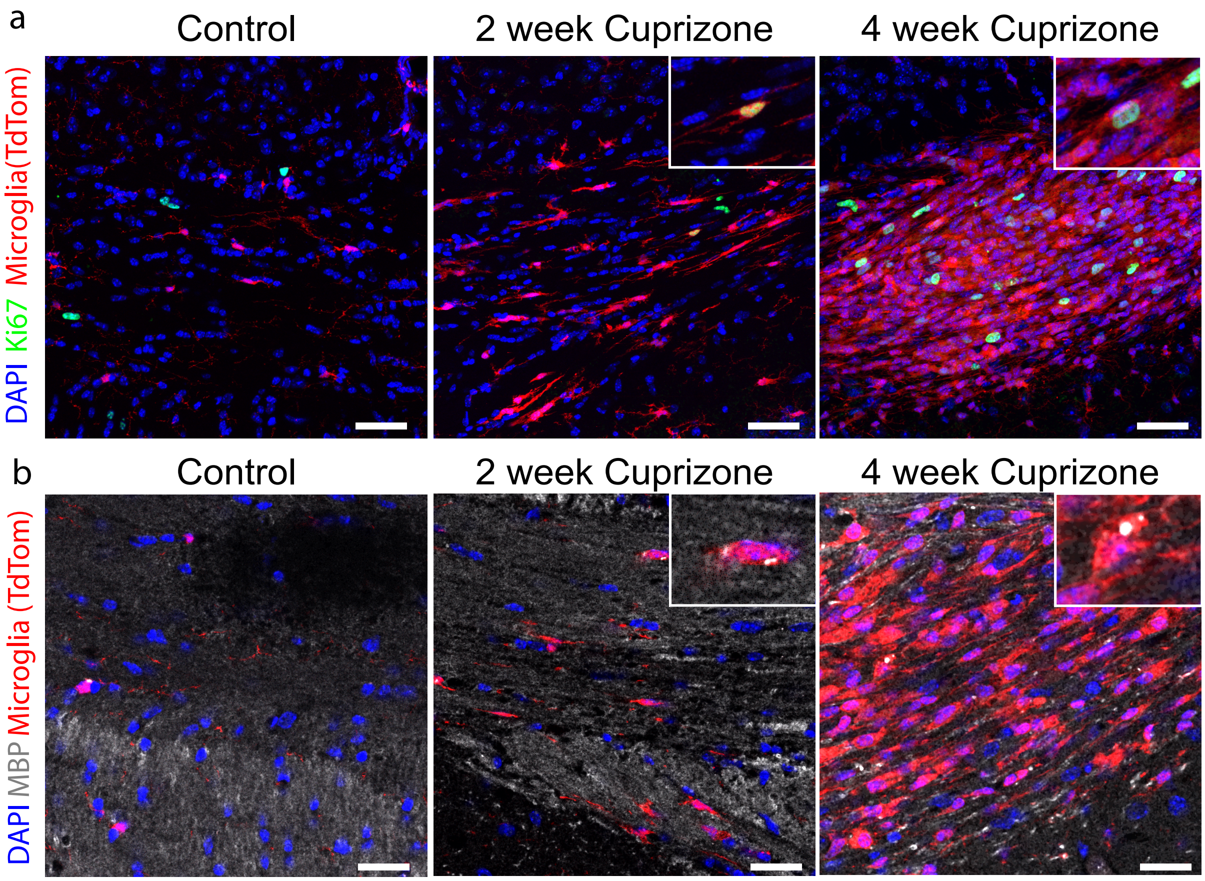

Supp Fig 2. Microglia proliferate and phagocytose myelin debris during cuprizone-induced demyelination. (**a**) Representative images of microglia expressing tdTomato (tdTom, red) in CX3CR1^{creER};Rosa^{tdTom} mice express the proliferative marker (Ki67) at 2 weeks and 4 weeks after the start of dietary cuprizone. (**b**) Representative images of microglia (tdTomato-positive cells) that contain the major myelin protein, myelin basic protein (MBP). The MBP aggregates inside of microglia was consistent with microglial phagocytosis of myelin debris. Scale bar, **(a)** 40µm, (b) 20µm.

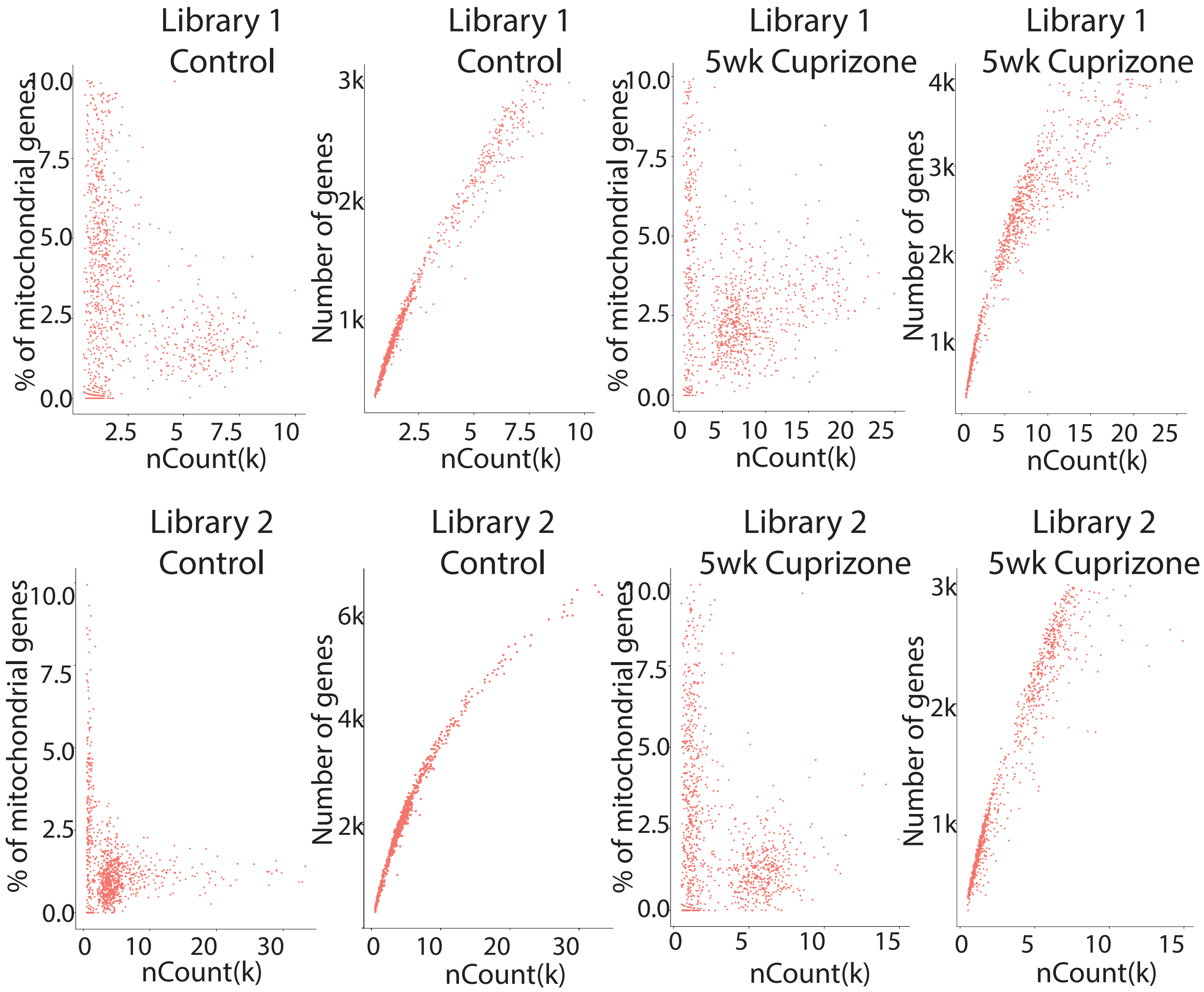

Supp Fig 3. Single-cell RNA sequencing quality control data. For quality control the proportion of mitochondrial genes and the number of genes were plotted against total number of molecules present in each cell (nCount) in thousands , for each library of control and 5 week cuprizone experiments.

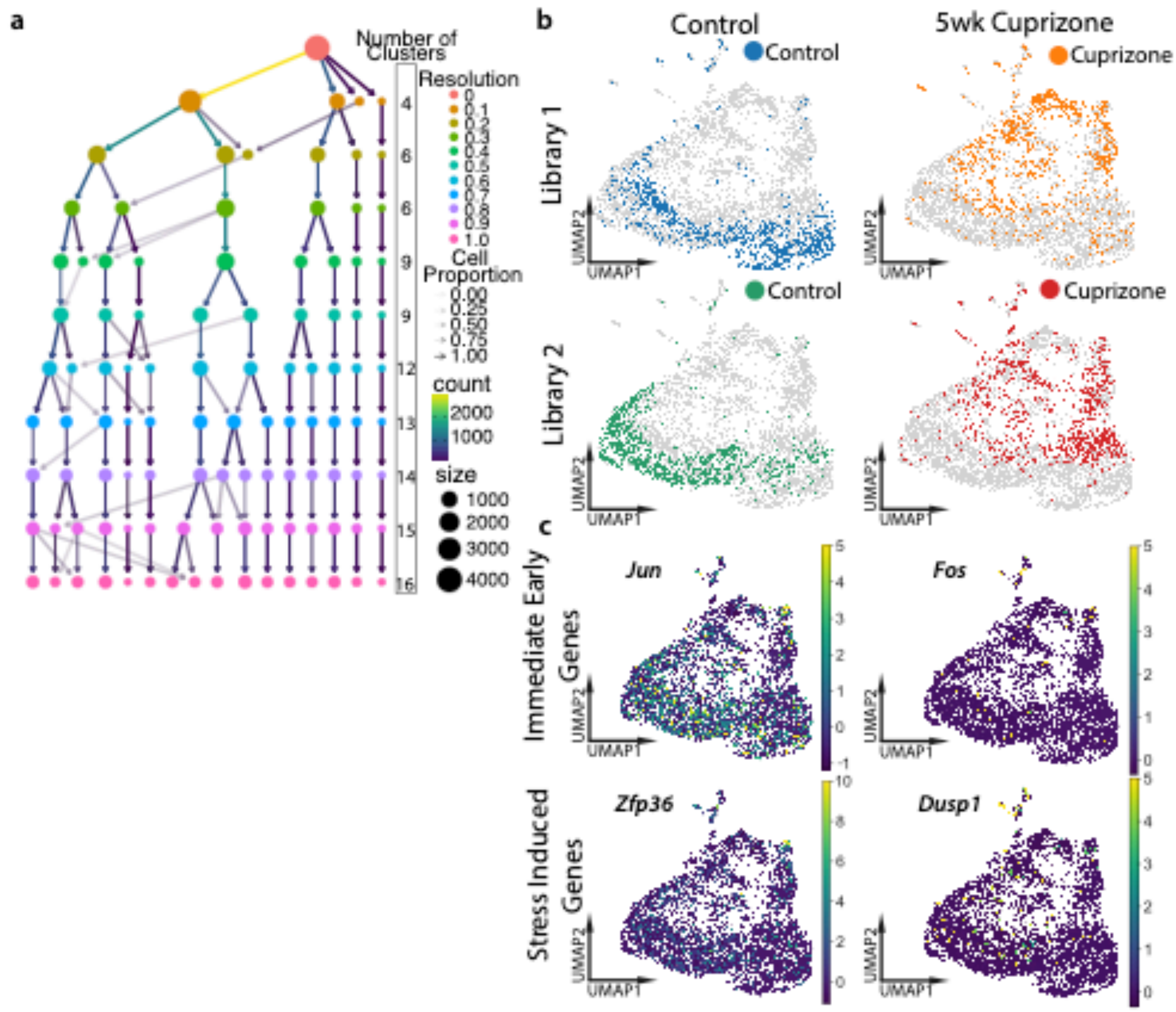

Supp Fig 4. Single-cell RNA sequencing approaches and validation. (**a**) A Clustree plot was used to determine optimal resolution. As resolution increases, moving down the clustering tree plot from 0 to 1.0, there is an increase in the number of clusters. With more clusters, there are fewer cells in each cluster (represented by circle size). As clusters split, arrows show the movement of cells from one cluster into multiple new clusters; the proportion of cells is indicated by arrow opacity. We chose resolution (0.5), where the mixing of cells is minimal. (**b**) Unsupervised graph-based clustering of individual libraries used in the larger dataset projected onto a UMAP. Control cells from two independent experiments, Library 1 (blue) and Library 2 (green), were primarily interspersed in the same regions. Likewise, cuprizone cells from Library

1 (orange) were largely interspersed with cuprizone cells from Library 2 (red), suggesting minimal batch effects. (**c**) Immediate early genes (*Jun* and *Fos)* and stress-induced genes (*Zfp36* and *Dusp1*) were not stimulated or concentrated in microglial clusters. Legend represents umi score with yellow representing a higher score.

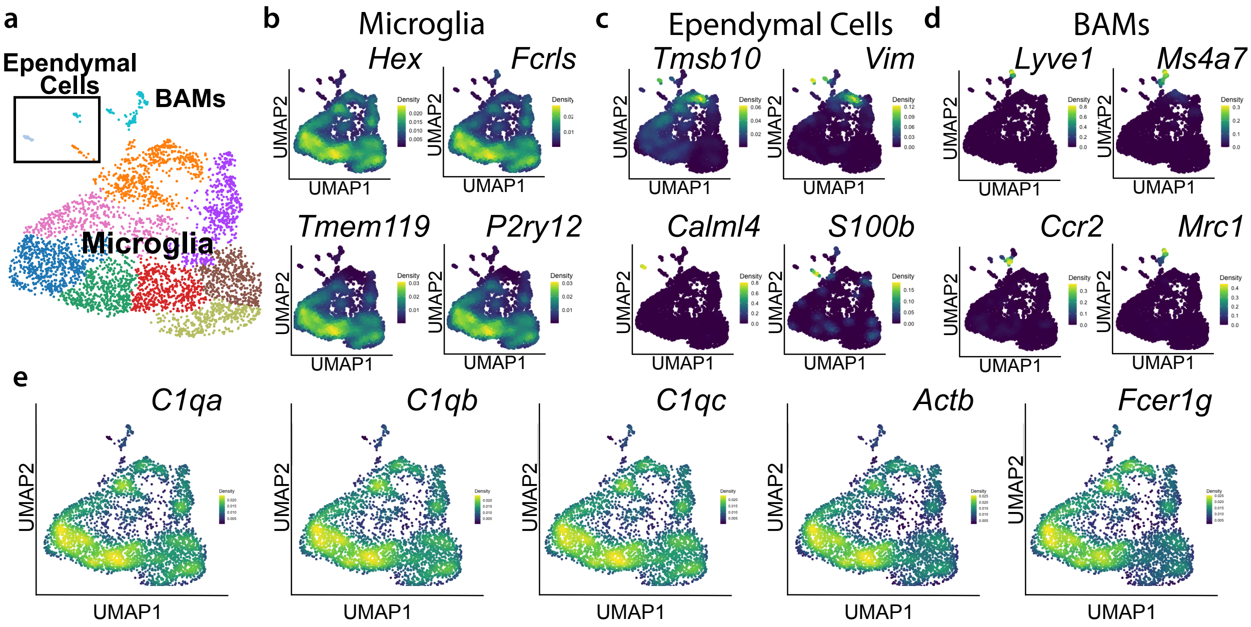

Supp Fig 5. Single-cell RNA sequencing annotation and homeostatic gene expression shift. (**a**) Unsupervised graph-based clustering and machine learning-based reclustering of dataset projected onto a UMAP. Cells were annotated guided by markers for (**b**) microglia, (**c**) Ependymal cells, and (**d**) border-associated macrophages (BAM). (**e**) Homeostatic microglia (control cell) subpopulations were located at the bottom of the microglial UMAP. We identified gene expression of complement protein genes (*C1qa, C1qb, C1qc*) and cell motility (*Actb* and *Fcer1g*) that were enriched within some homeostatic microglia subpopulations (microglia, bottom left) but not others (microglia, bottom right). Density represents the kernel density estimation, which resolves gene expression sparsity and rescues drop-out genes based on neighbouring clusters.

Supp Fig 5

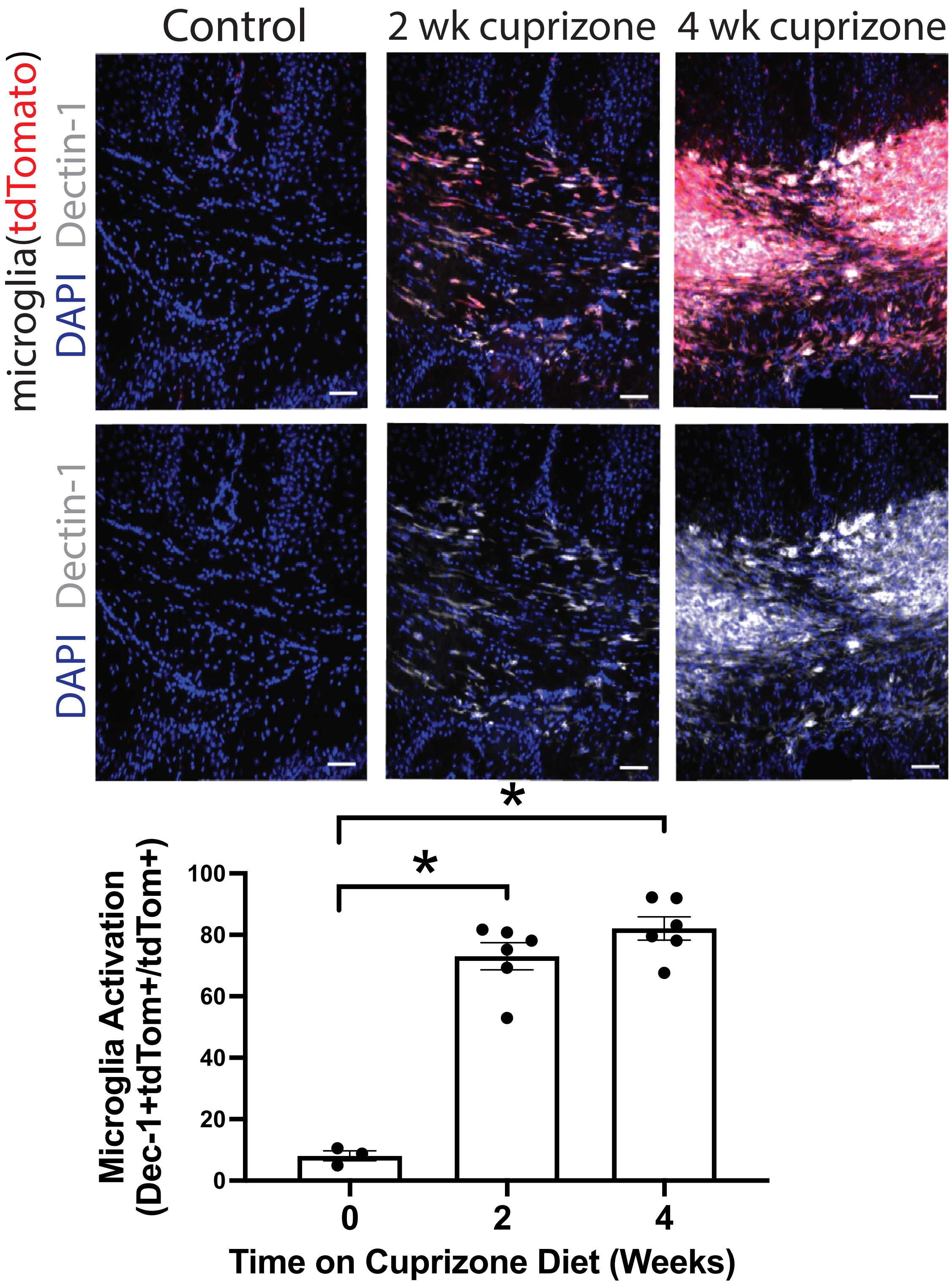

Supp Fig 6. Dectin1/Clec7a was enriched within microglia within two weeks after initiating the cuprizone diet. Quantification and representative immunohistochemical images demonstrating

microglia (tdTomato) express Dectin1 at two and four weeks after initiating the cuprizone diet. n=3 for control and n=6 for 2 and 4 weeks post. One-way ANOVA with Tukey's multiple comparison test. Error bar ± SEM. Scale bar, 50μm.

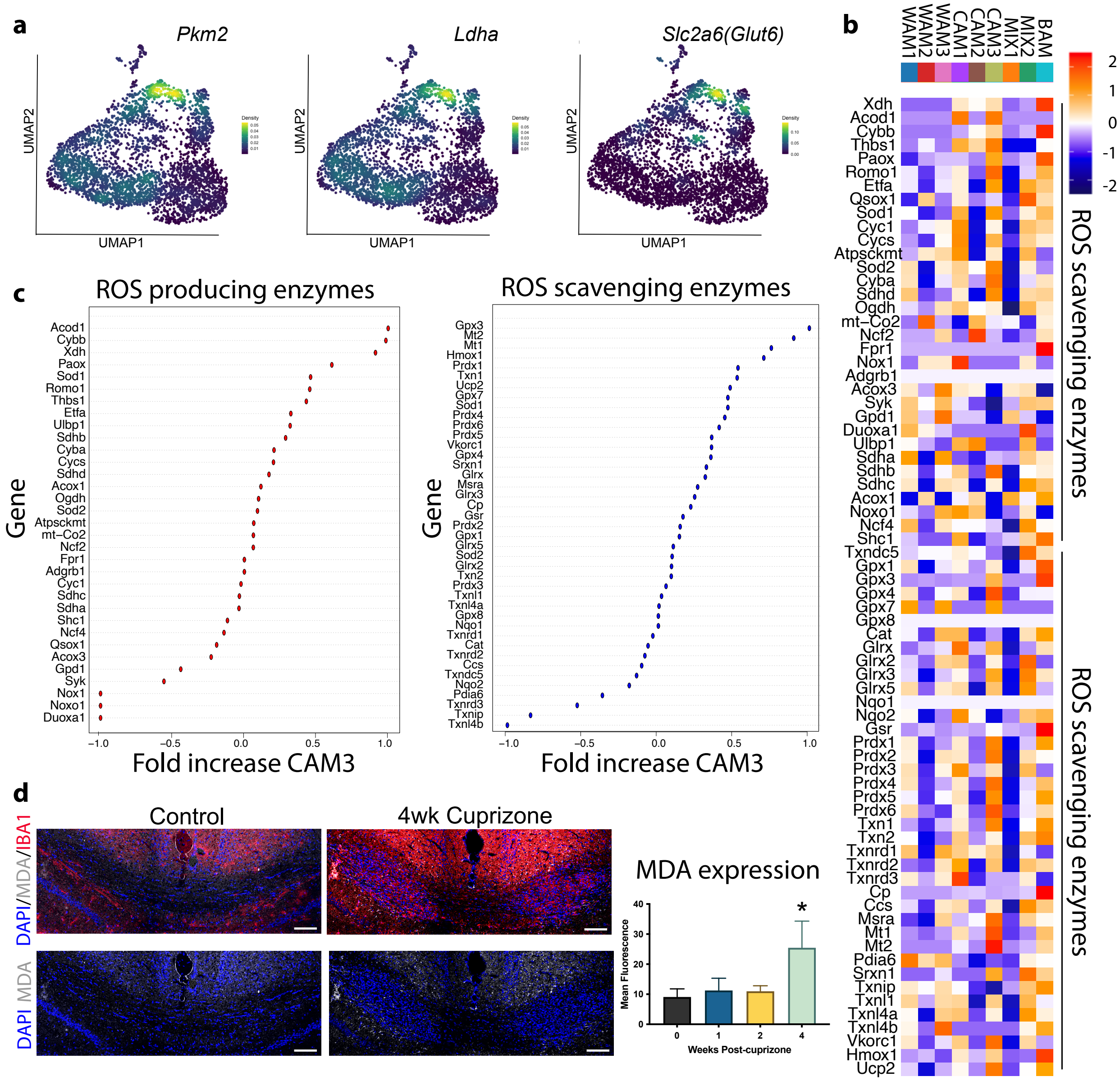

Supp Fig 7. Cuprizone-associated microglia gene transcriptional enrichment in glycolysis and reactive oxygen species (ROS) production. (**a**) Microglia and BAM clusters projected onto a UMAP show enrichment of genes related to glycolysis (*Pkm2, Ldha*) and glucose transport (*Glut6*, or *Slc2a6*). Density represents the kernel density estimation, which resolves gene expression sparsity and rescues drop-out genes based on neighbouring clusters. (**b**) ROS producers and scavengers plotted on a heat map with normalized gene expression derived from

cluster identity. (**c**) Fold change with ROS producing and scavenging enzymes. Fold change was calculated with averaged gene expression for [(CAM3-WAM1)/(CAM3+WAM1)]. (**d**) Quantification and representative immunohistochemical images demonstrating lipid peroxidation (Malonaldehyde, or MDA, white) located within regions associated with microglia (IBA1, red) accumulation at four weeks after initiating cuprizone diet. n=5-7 with One-way ANOVA with Dunnett's multiple comparison test. Error bar ± SEM. Scale bar, (**d**) 100μm.

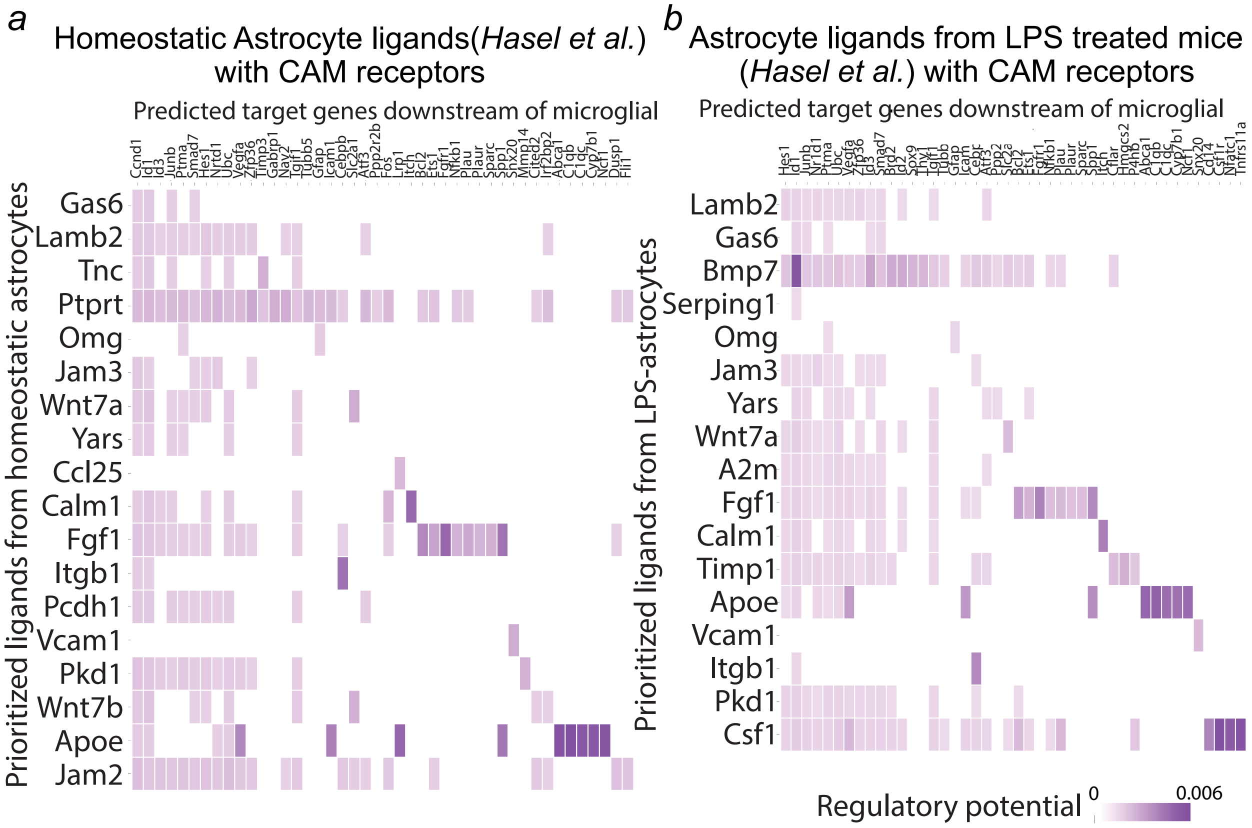

Supp Fig 8. Potential astrocyte factors that regulate the cuprizone associated microglial (CAM) state. We used NicheNet to compare (**a**) homeostatic astrocytes and (**b**) astrocytes from LPS-treated mice from Hasel and colleagues' datasets[37] with CAM. NicheNet compared known secreted factors from these astrocyte datasets with CAM expressed receptors, but also examined the downstream genes regulated by these receptor systems. NicheNet calculates a regulatory

potential for CAM genes based on known receptor-ligand interactions and identified transcriptomic expression levels. Naive astrocyte ligands such as *Apoe* and *Fgf1,* and LPS-stimulated astrocyte ligands like *Bmp7, Fgff1, ApoE*, and *Csf1*were identified as potential regulators of the CAM state.

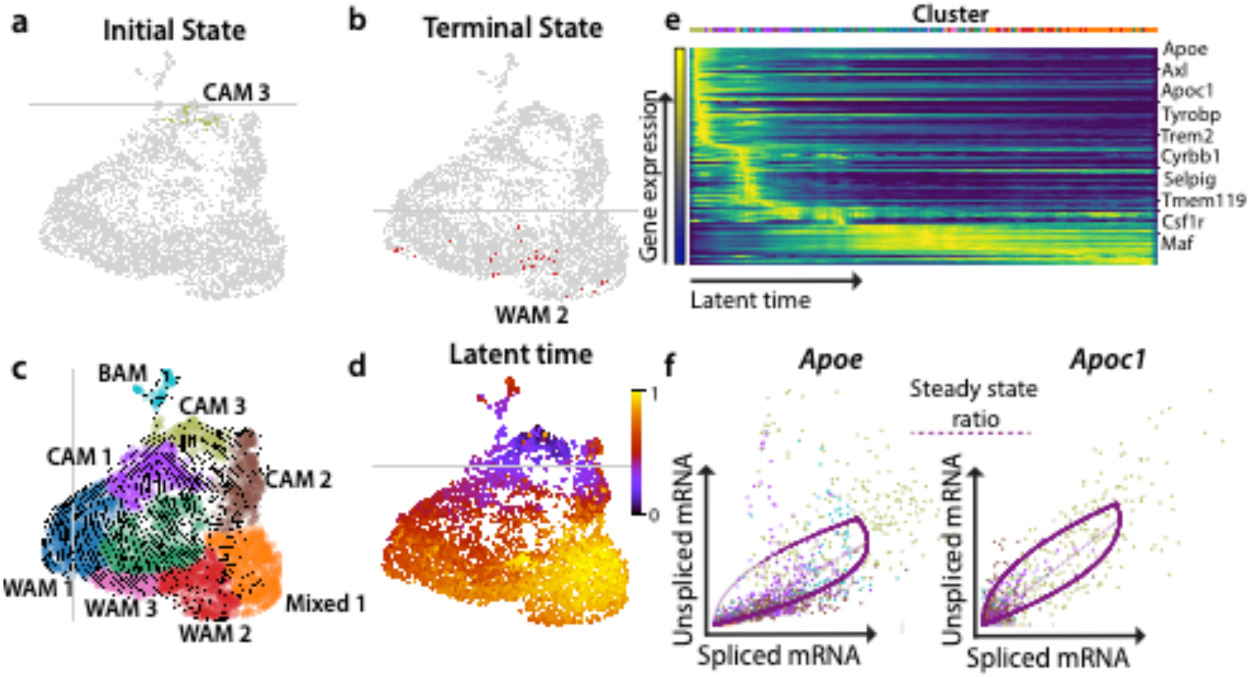

Supp Fig 9. Through trajectory analysis, cuprizone-associated microglia (CAM) subpopulations are linked to white matter-associated microglia (WAM) subpopulations. Cell Rank was used to identify the (**a**) initial and (**b**) terminal predicted states. (**c**) ScVelo was used to determine the cell path and trajectory of cells proceeding from one subpopulation to another. (**d**) Latent time describes the probability of being at an initial state (0, purple) or terminal state (1, yellow). (**e**) A gene expression heat map shows those genes that increase as cells transition from different states across latent time. Legend represents gene expression, with yellow representing a higher value and blue depicting a lower value. (**f**) CellRank was used to identify potential driver genes or genes involved in a fate choice between different states. We interrogated potential driver genes involved in the transition between CAM3 and other microglial states to identify the genes

Apoe and *Apoc1*. These genes were plotted such that each dot represented a cell whose color provides cluster identity. The cell is plotted based on the extent of unspliced vs. spliced mRNA to depict RNA velocity.

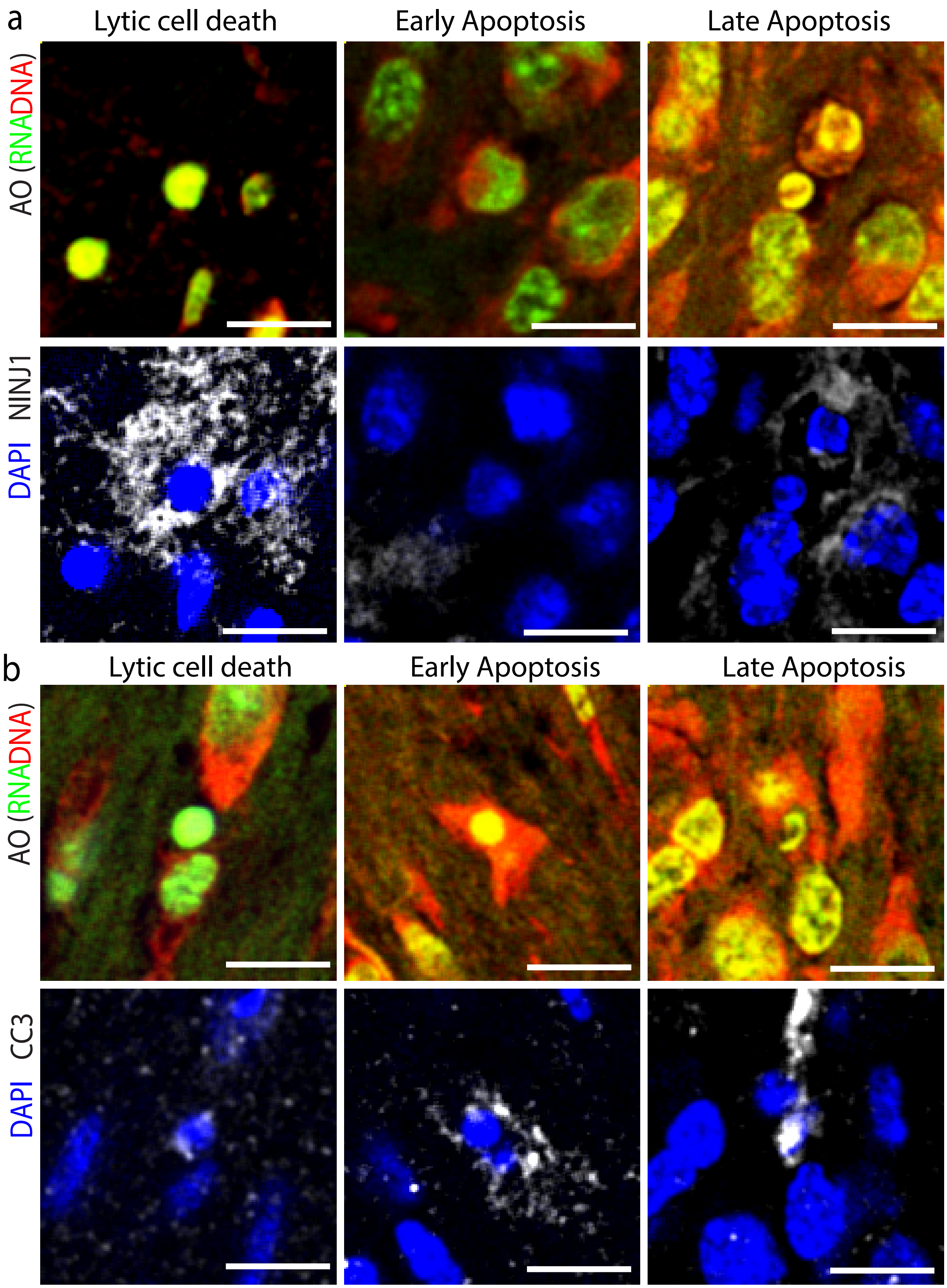

Supp Fig. 10 Acridine orange (AO) based cell death morphologies compared to membrane lysis protein Ninjurin-1 (NINJ1) and apoptosis protein product cleaved caspase-3 (CC3). (**a**)

Representative images of cell death based on AO morphologies that were lytic, early apoptotic, and late apoptotic and their accompanying expression of NINJ1, a marker of plasma membrane rupture. We found prominent expression of NINJ1 in lytic cells, but limited expression within cells with apoptotic morphologies. (**b**) Representative images of lytic, early apoptotic, and late apoptotic cell populations and the corresponding expression of cleaved caspase-3 (CC3), an apoptosis marker. Cells with apoptotic morphologies often expressed CC3, but cells with lytic morphologies expressed little to no CC3 expression. Scale bars, (a, b) 10μm

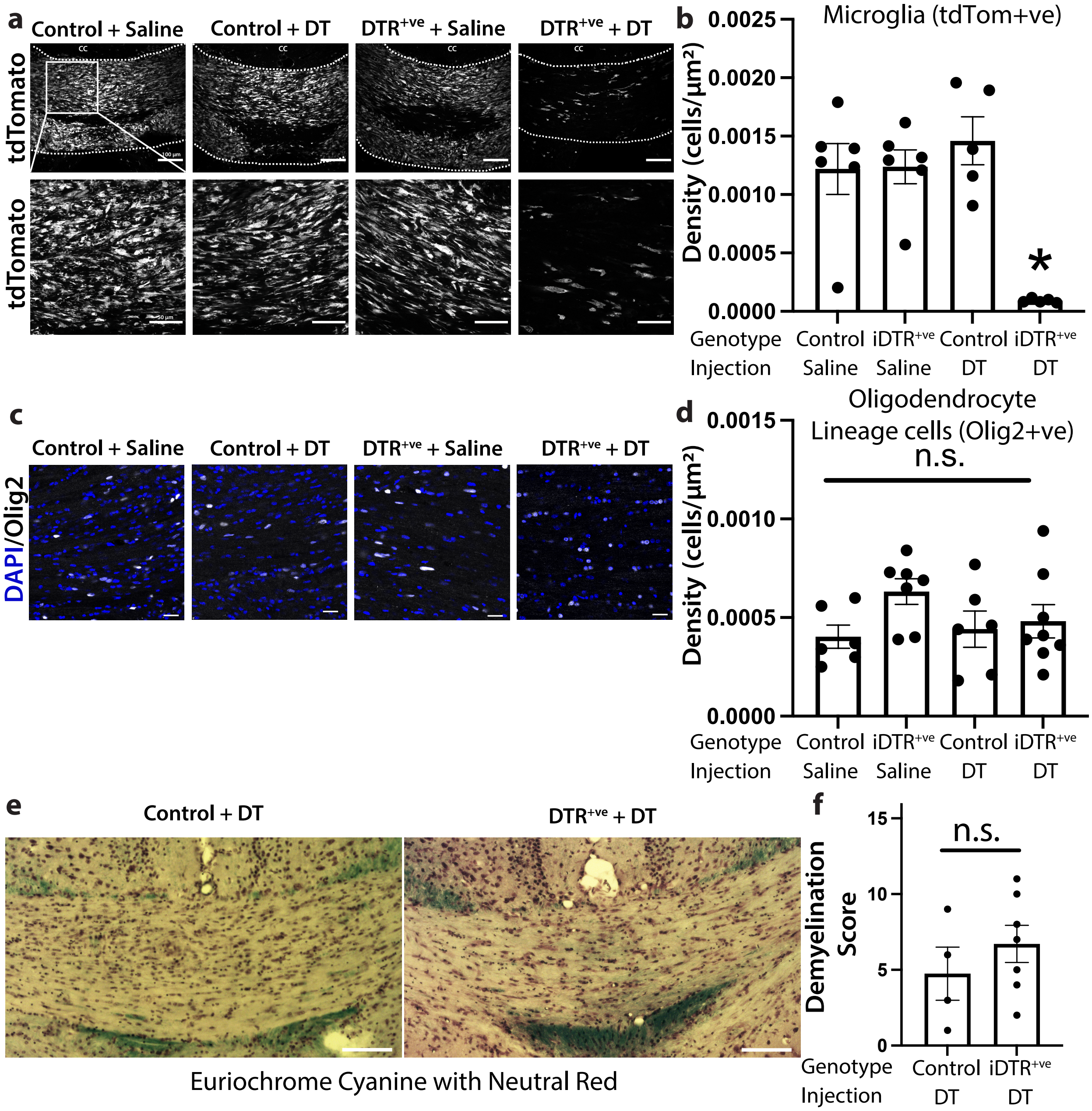

Supp Fig 11. Microglial ablation two weeks after mice are placed on dietary cuprizone does not alter demyelination. (**a, b**) Representative immunohistochemical images and quantification demonstrating that microglia become ablated in mice expressing inducible DTR mice ($CX3CR1^{CreER}$; $Rosa^{iDTR}$) treated with diphtheria toxin (DT), but not in control $CX3CR1^{CreER}$ mice treated with saline (Control + Saline), $CX3CR1^{CreER}$ mice treated with DT (Control + DT), or $CX3CR1^{CreER}$; $Rosa^{iDTR}$ mice treated with saline (DTR^{+ve} + saline). (**a**) The top image was

taken with lower primary magnification (10x) compared to the lower image (40x). (**c, d**) Quantification and representative immunohistochemical images showing microglia ablation did not change oligodendrocyte lineage cell density (Olig2, white). (**e, f**) Representative widefield image of euriochrome cyanine and neutral red-stained sections with quantification showing no difference in demyelination between control CX3CR1^{CreER} with DT (control + DT) mice and CX3CR1^{CreER}; Rosa^{iDTR} mice treated with DT (DTR^{+ve} + DT). (B,D,F) n=4-7. (**b, d**) One-way ANOVA, (**f**) Unpaired T-Test. Scale bar, (**a**-Top, **e**) 100µm, (**a**-bottom) 50µm, (**c**) 20µm.

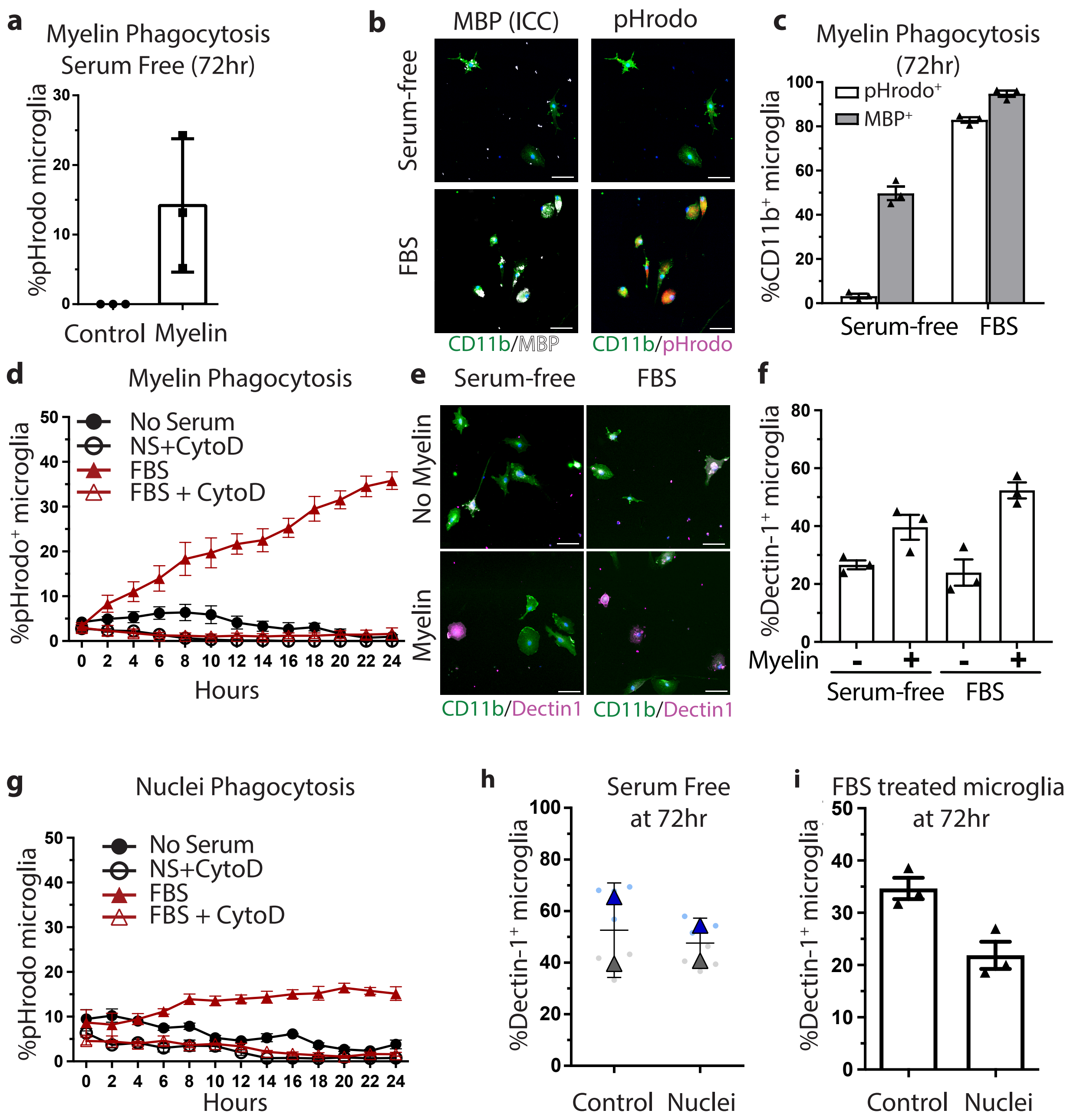

Supp Fig 12. Microglia phagocytosis of myelin and nuclei in a serum-free culture system. (**a**) There was limited phagocytosis of pHrodo labelled myelin by 72hr in serum-free microglial cultures. Representative images (**b**) and quantification (**c**) comparing pHrodo labelled myelin with the major myelin protein, myelin basic protein (MBP), following immunocytochemistry (ICC) demonstrates that serum increases myelin phagocytosis. (**d**) Based on pHrodo fluorescence, microglia phagocytosis was inhibited with cytochalasin D (CytoD). Representative

immunocytchemical image (**e**) and quantification (**f**) demonstrating myelin debris exposure increases the proportion Dectin1 expressing microglia, in purified, serum-free and serum-containing (FBS) cultures. (**g**) Microglia phagocytose pHrodo labelled nuclei when they are treated with FBS, but not under serum-free conditions or with the phagocytosis inhibitor CytoD. Neither the exposure to nuclei under serum-free (**h**) or FBS (**i**) conditions promoted more expression of the cuprizone-associated microglia marker Dectin1/Clec7a. (**h**) n=3 wells in 2 independent experiments or one (**a, c, f, i**) independent experiment. Error bar ± SEM. Scale bar, (***b, e***) 50μm.
